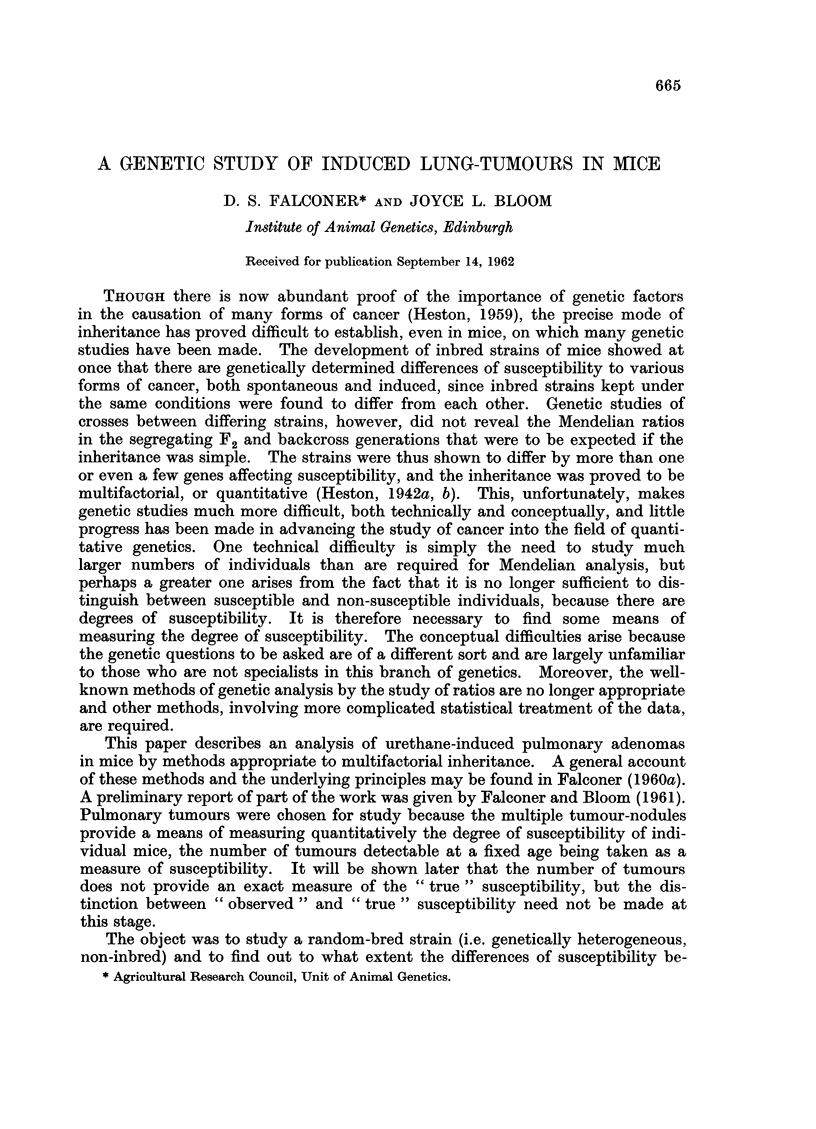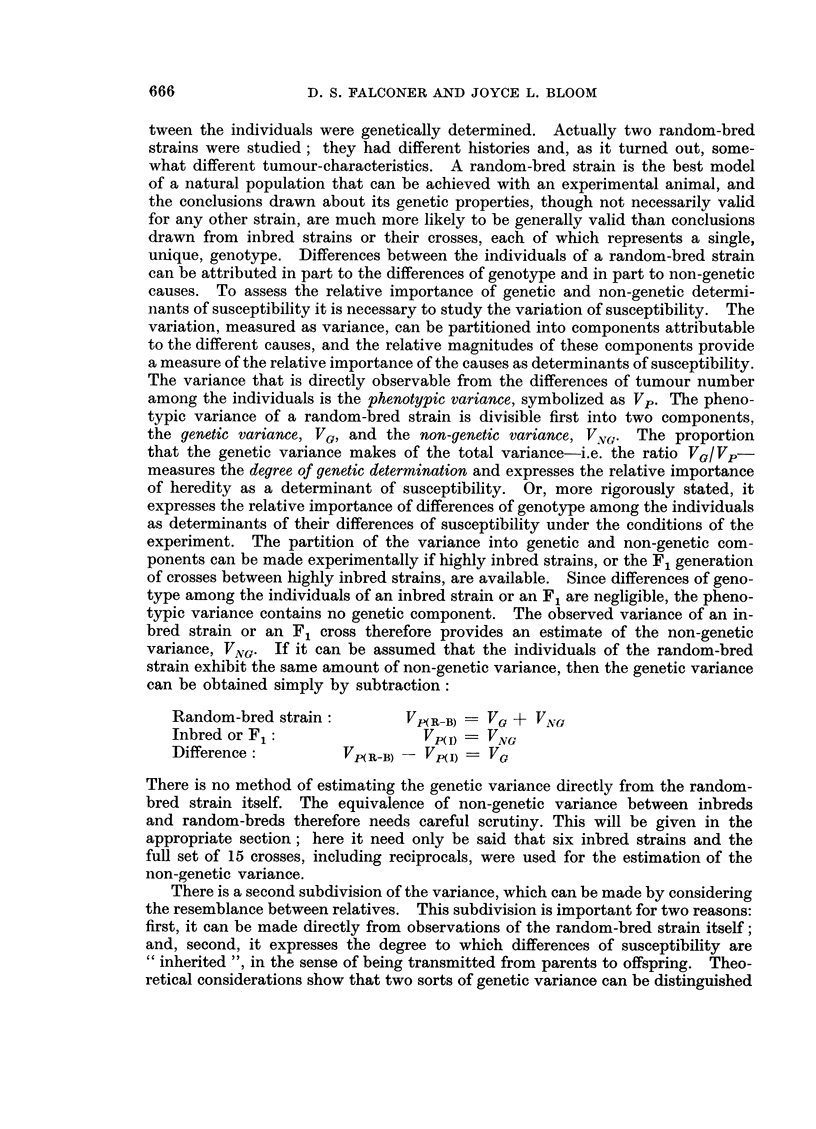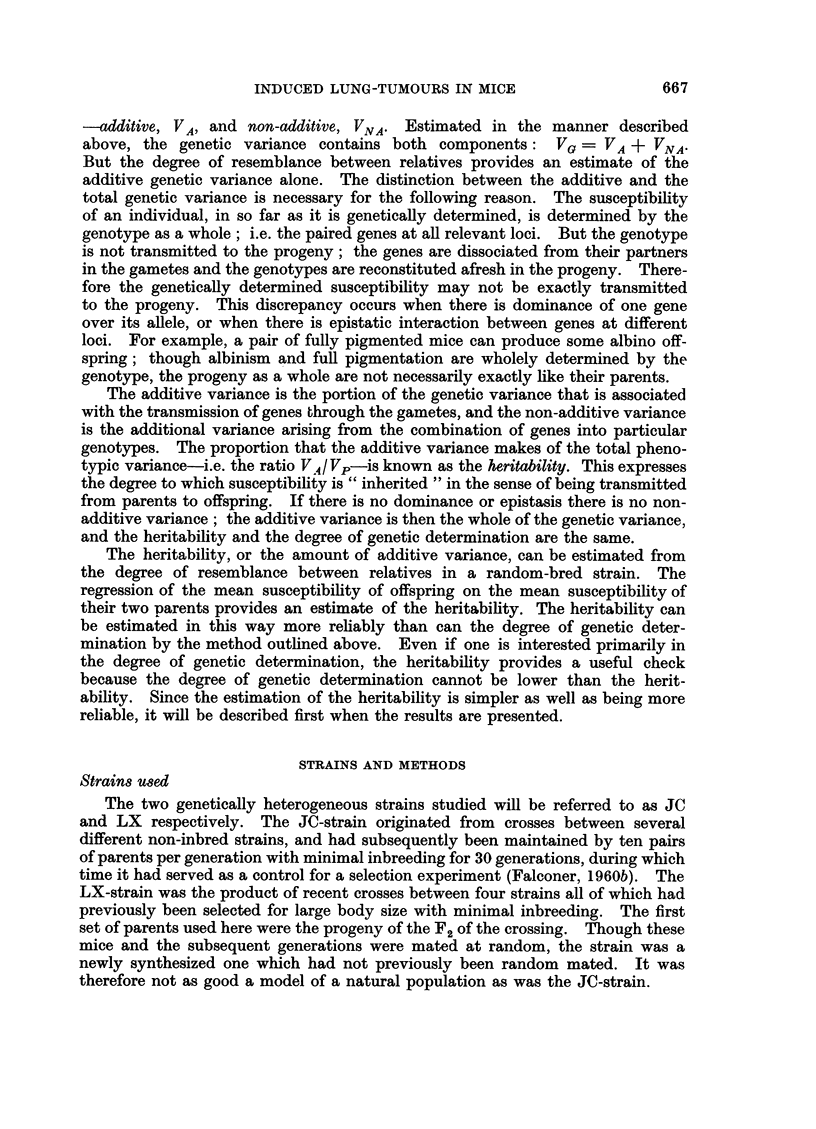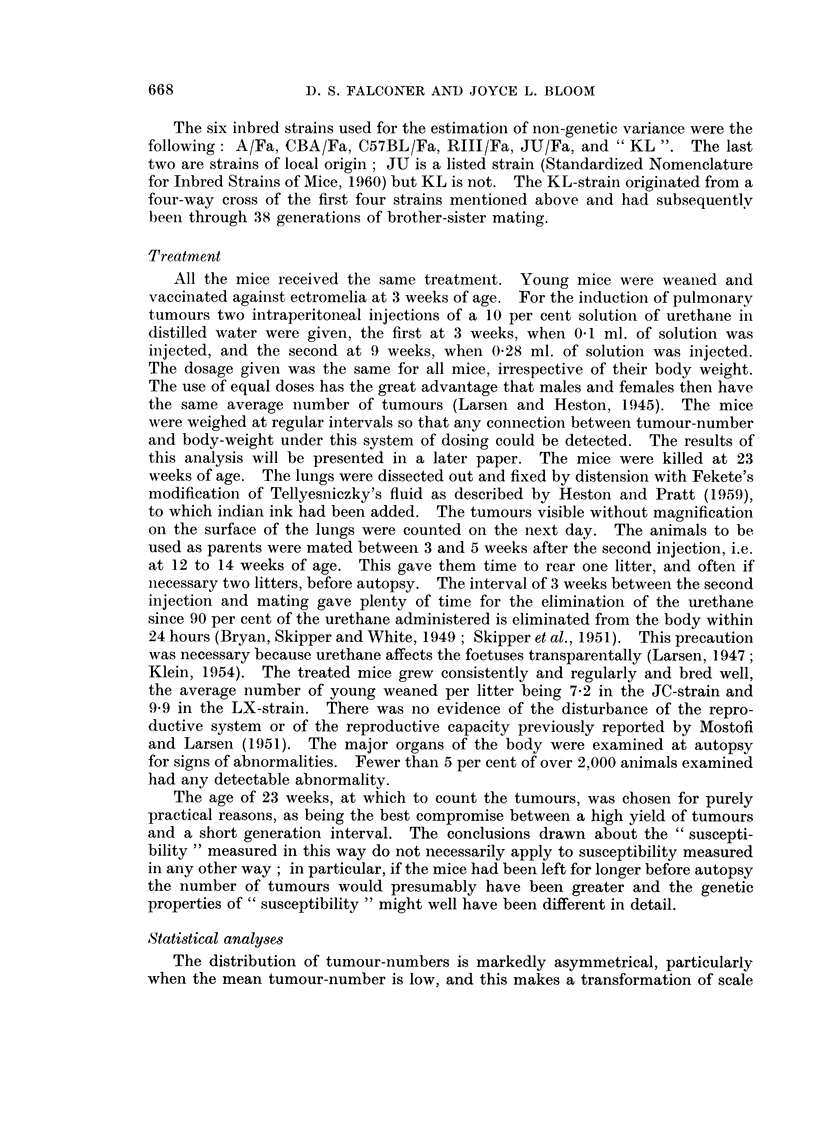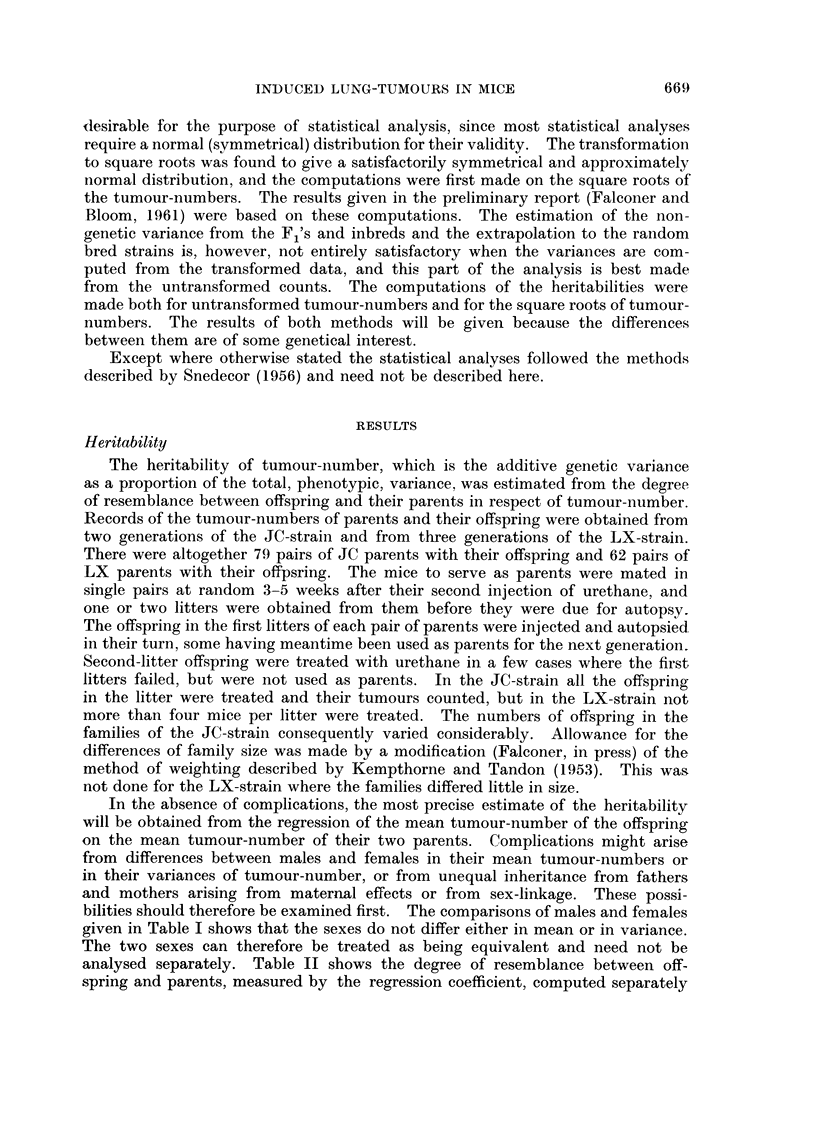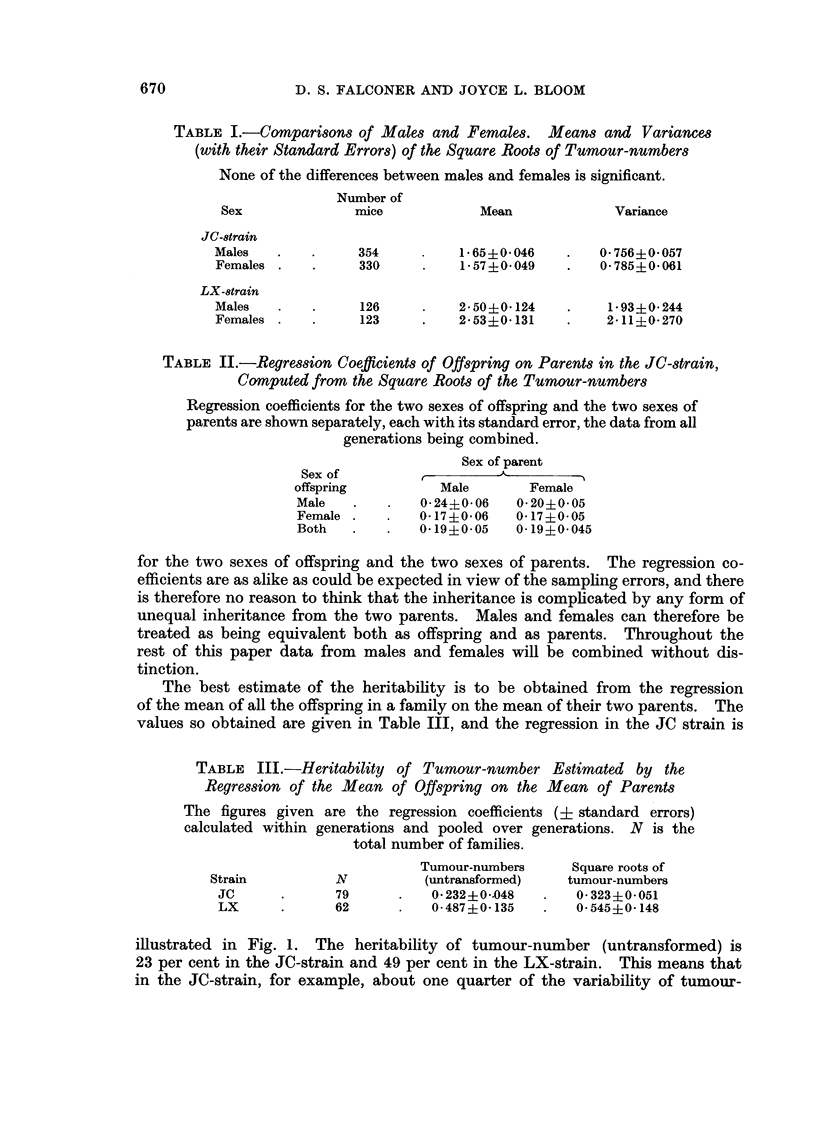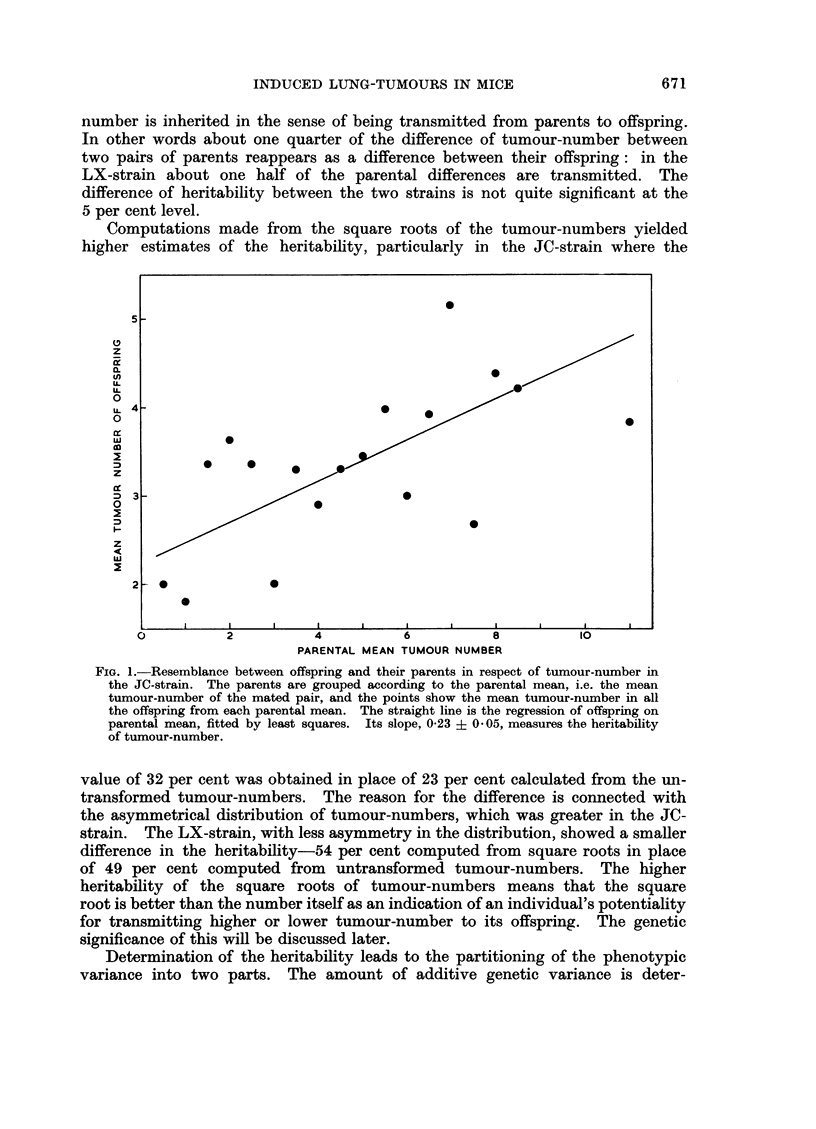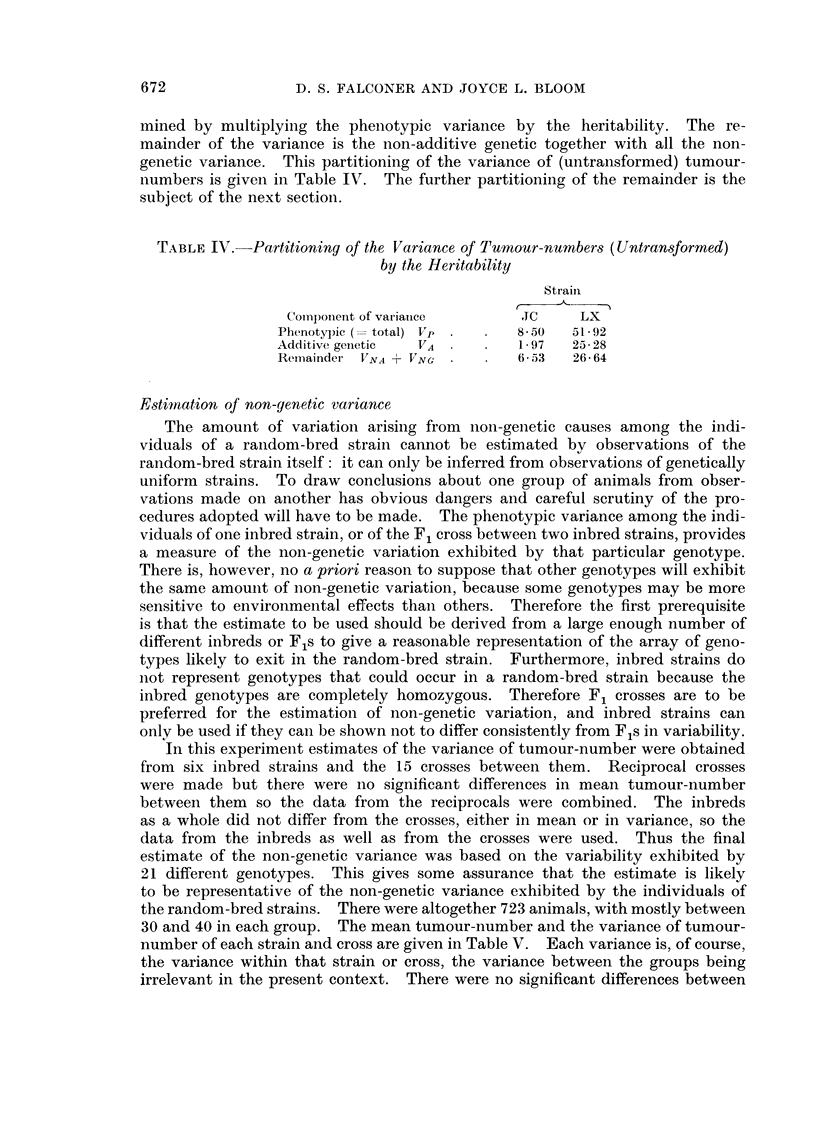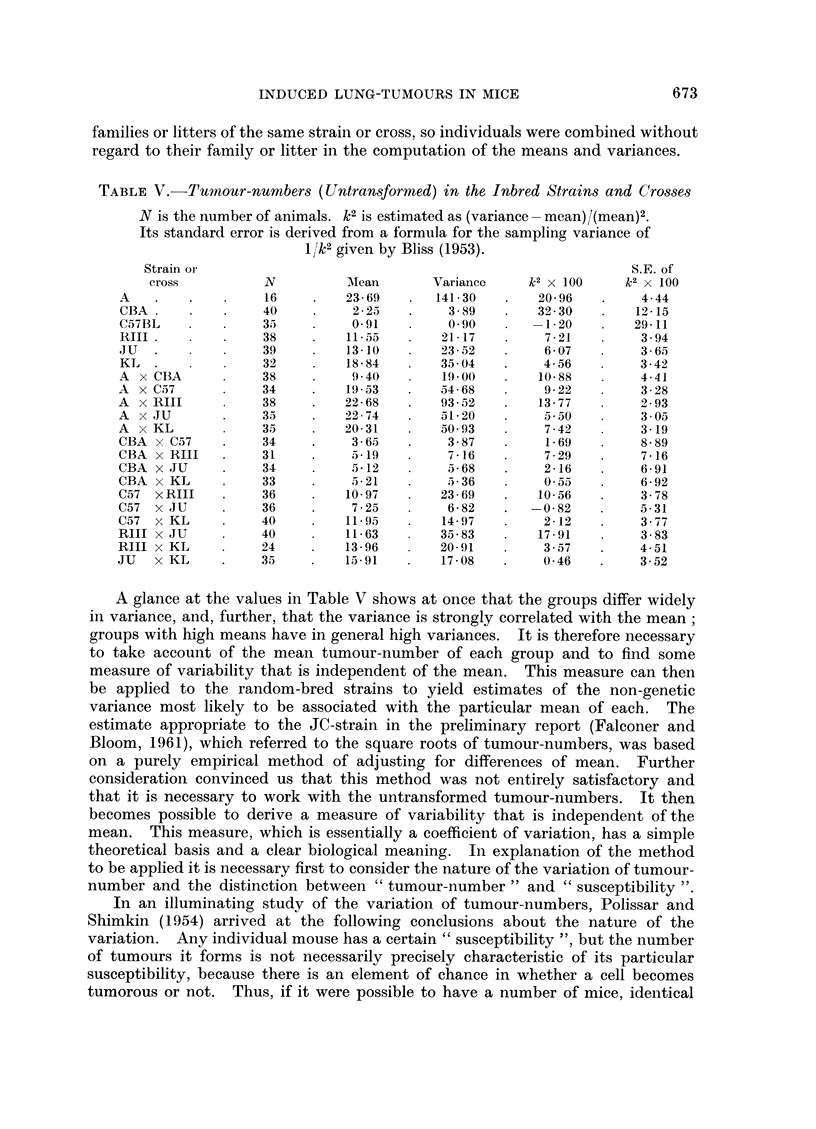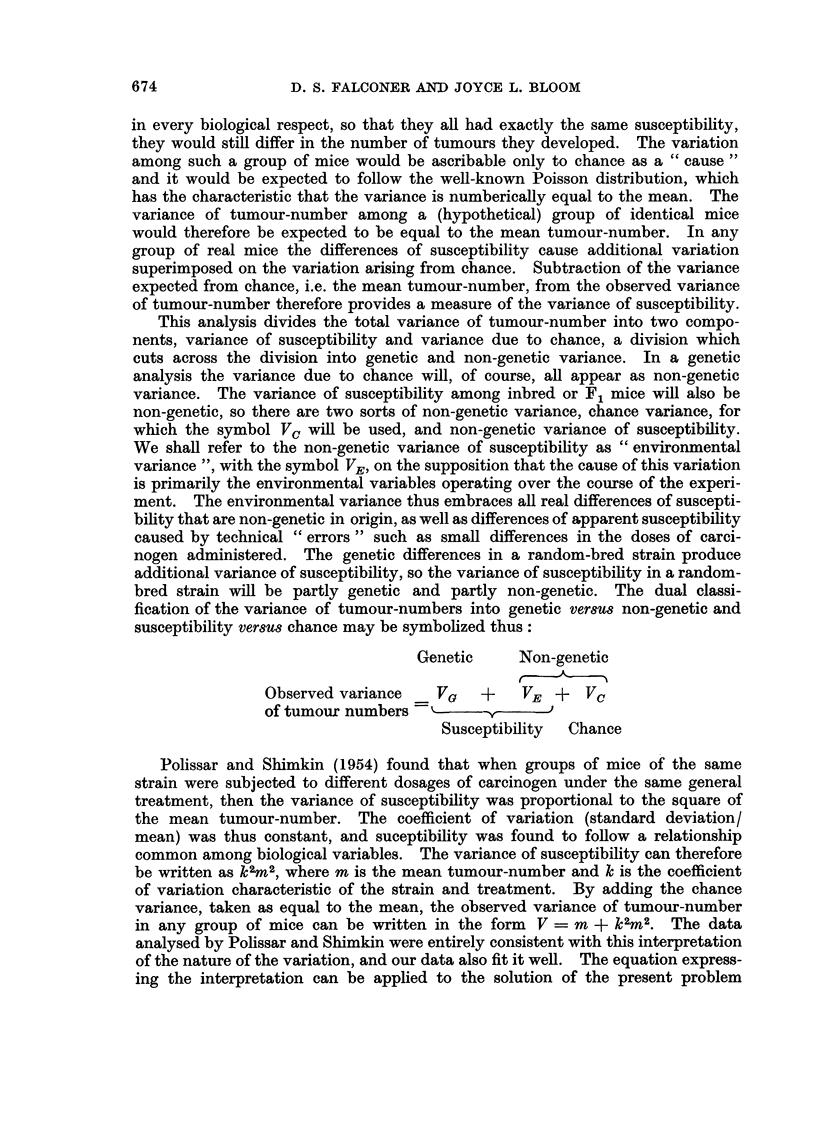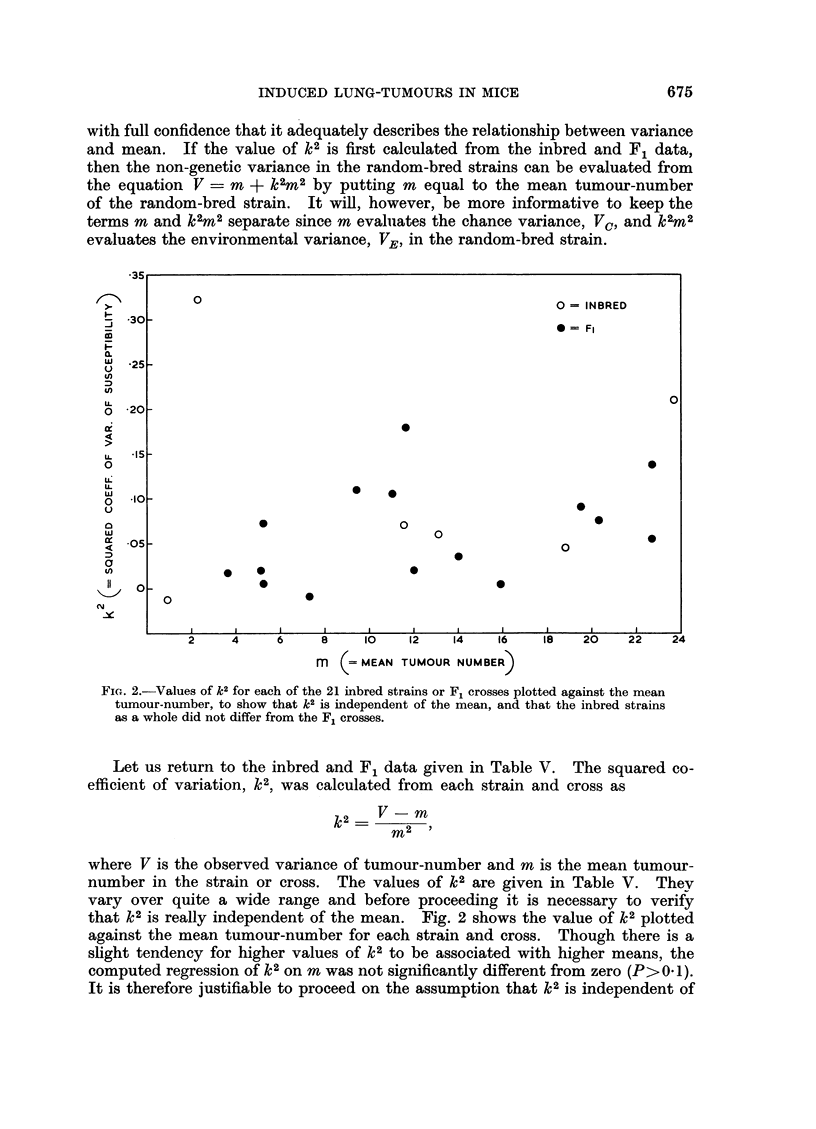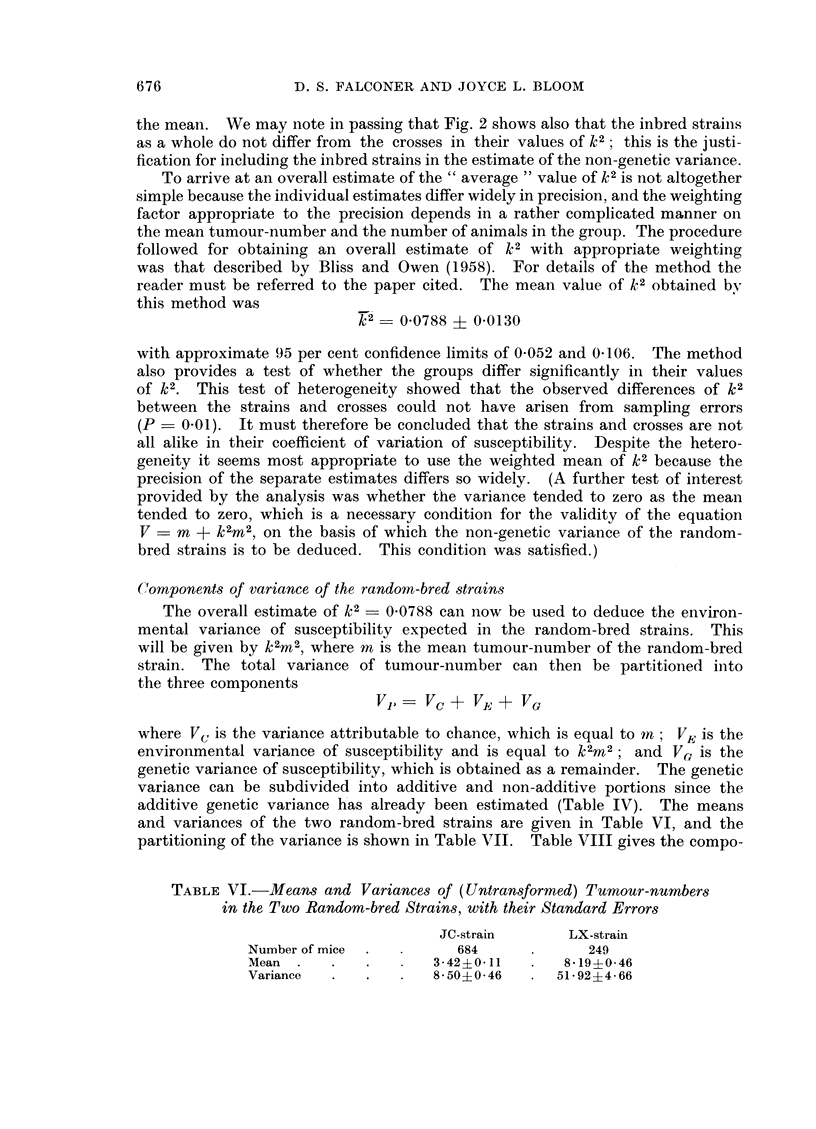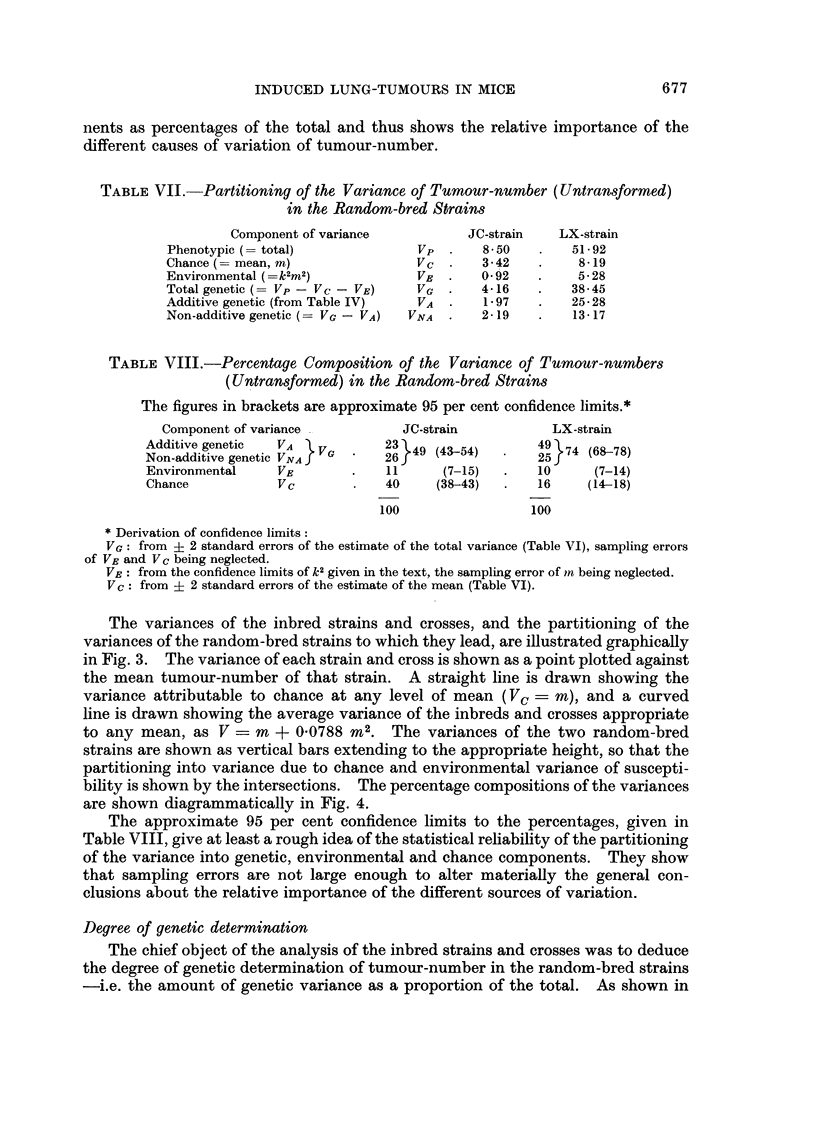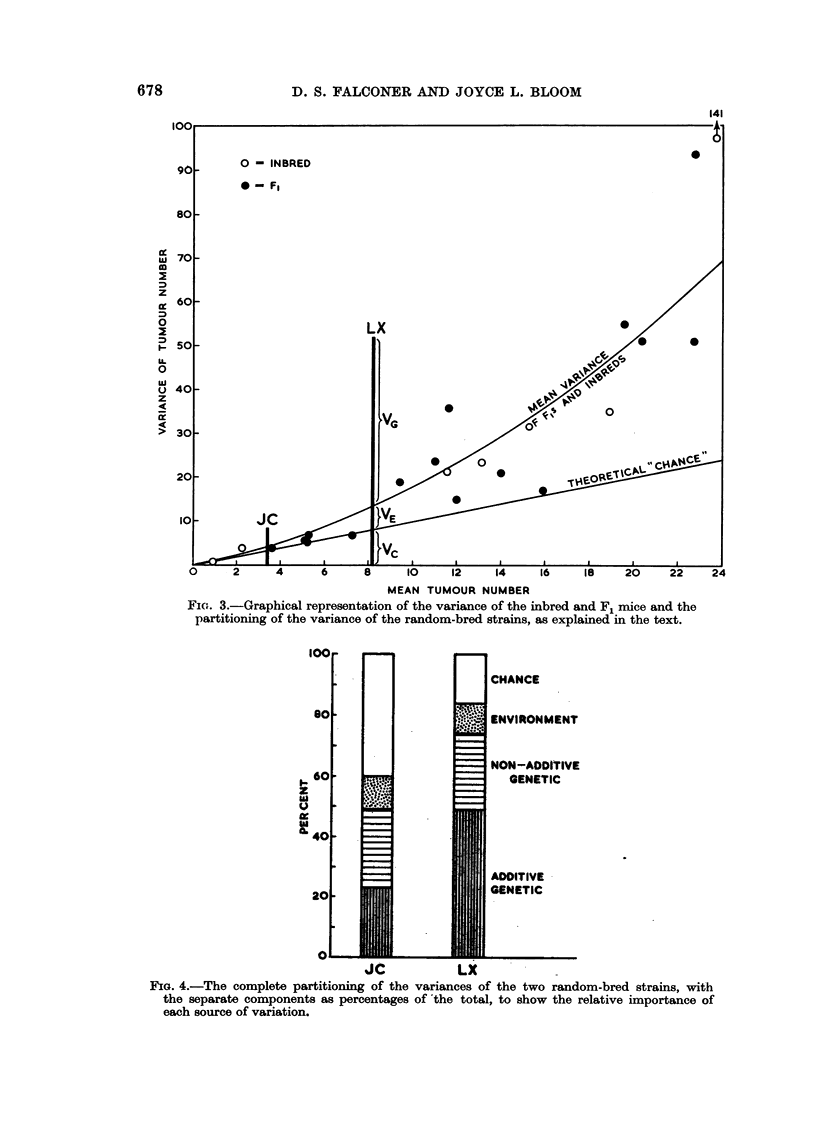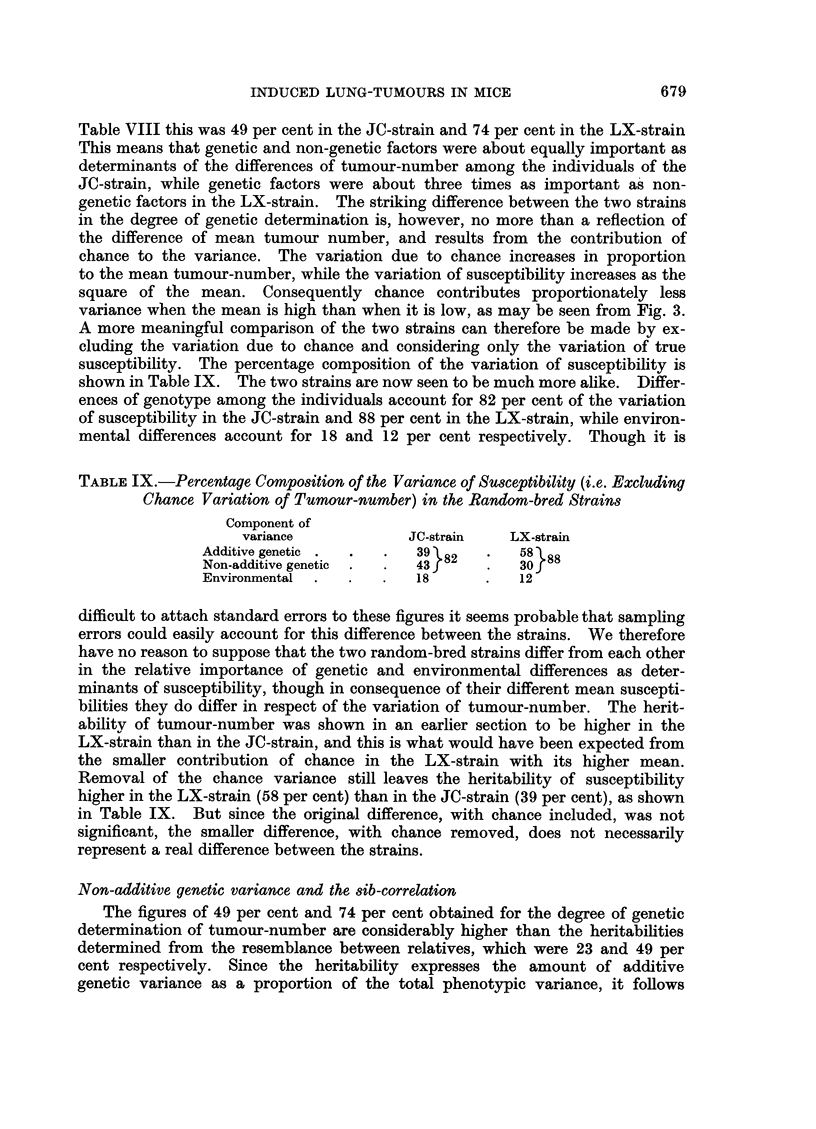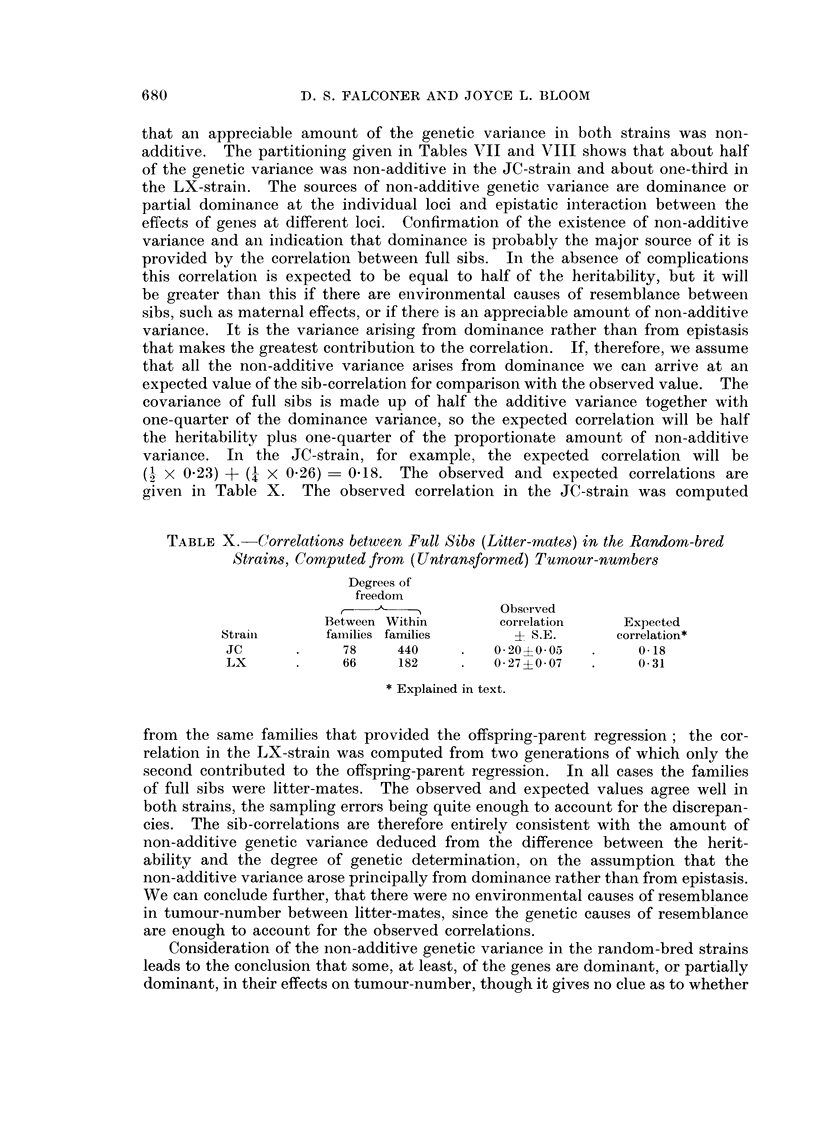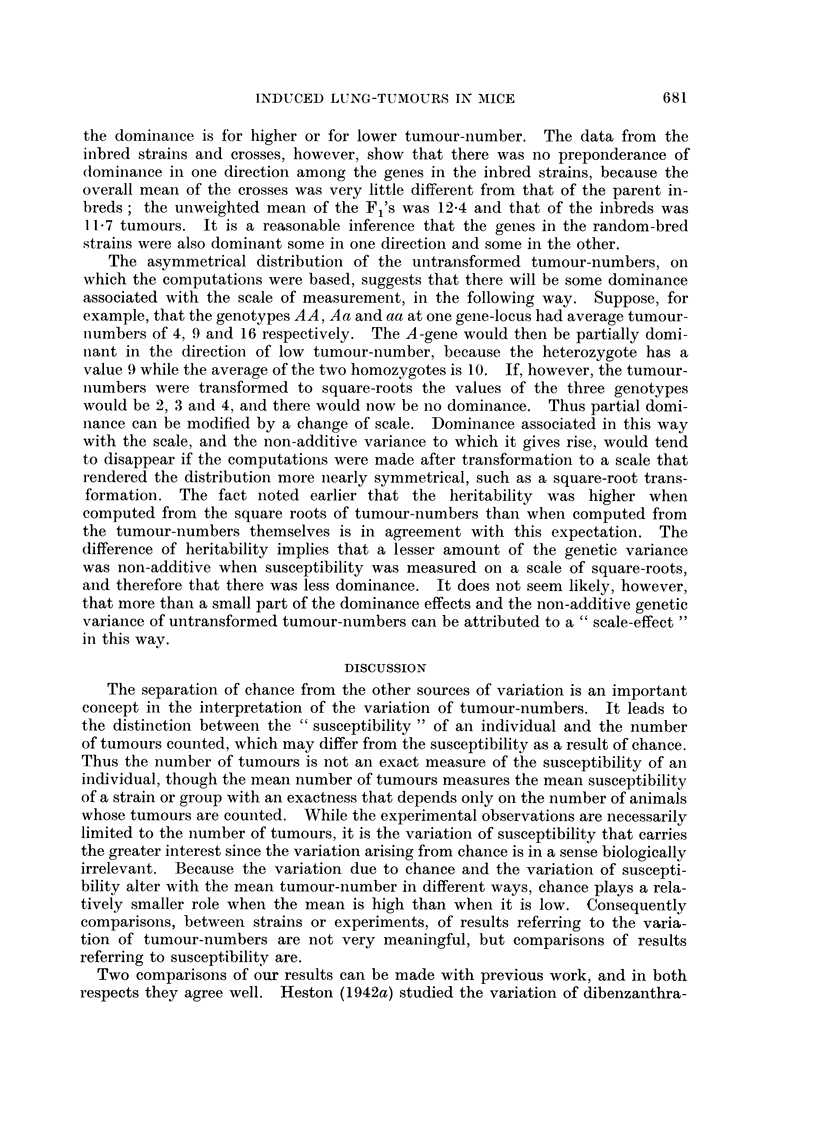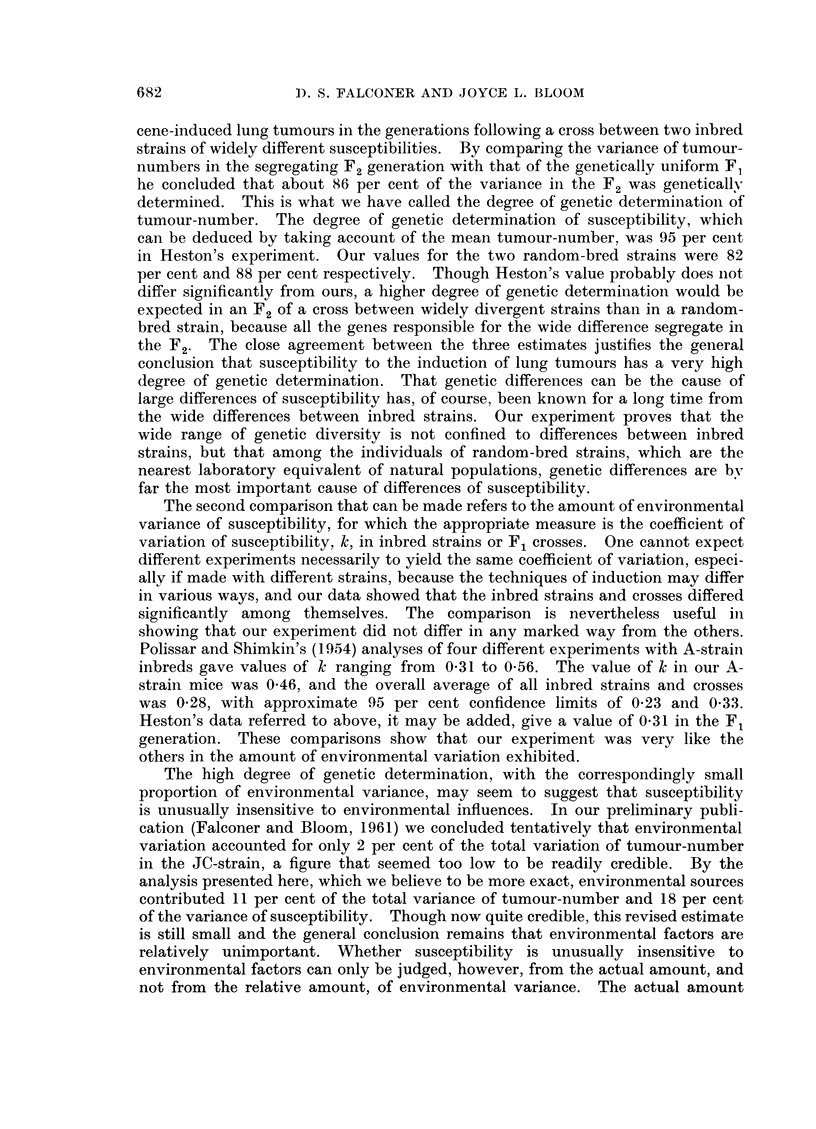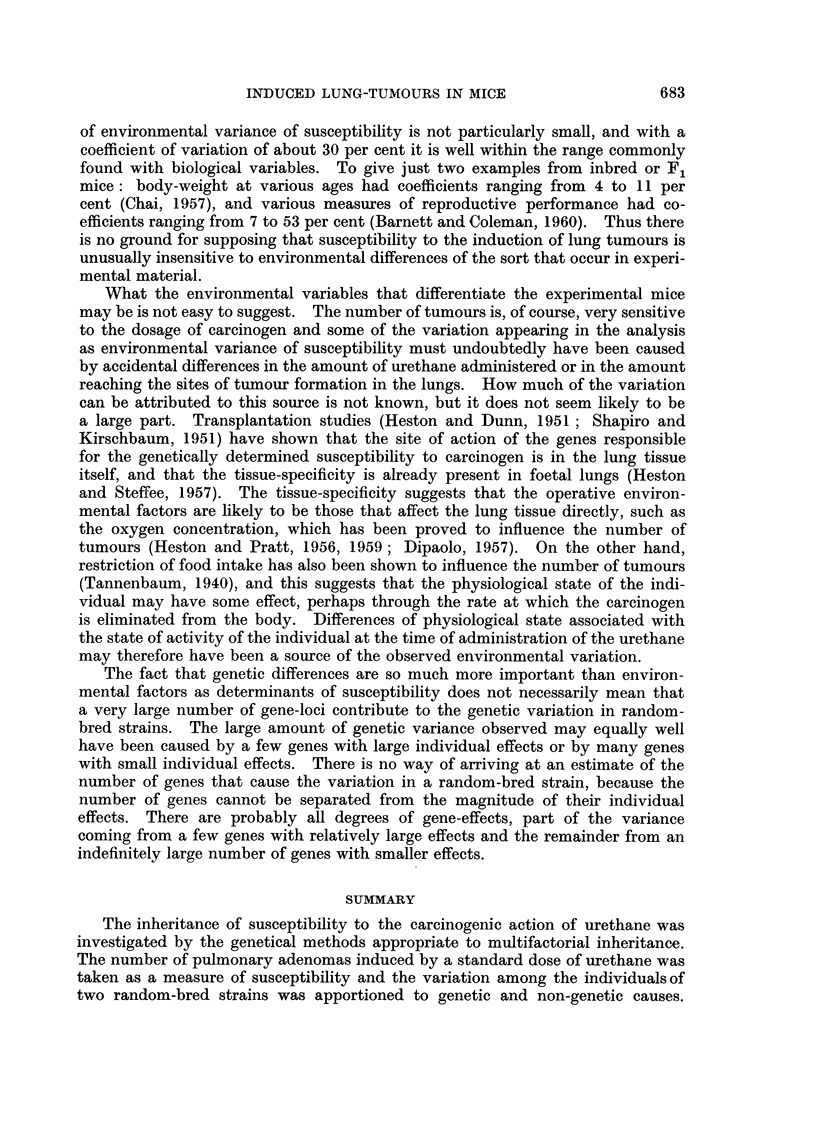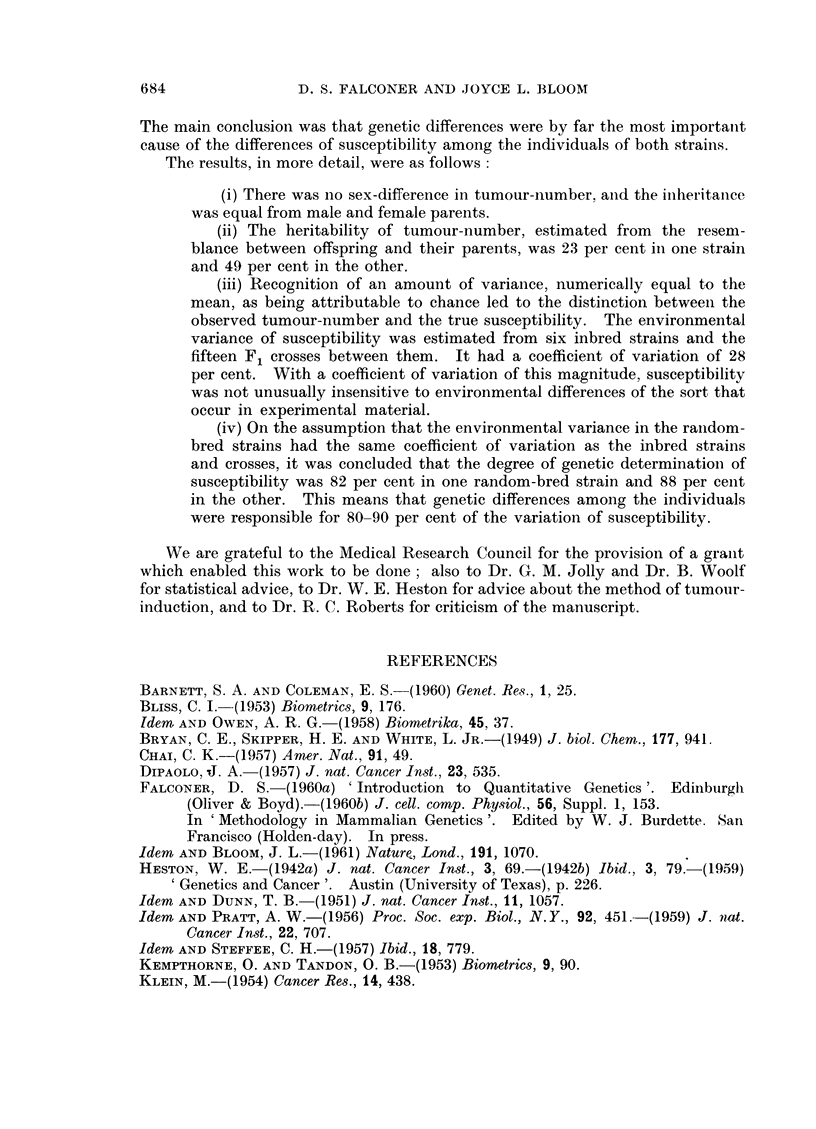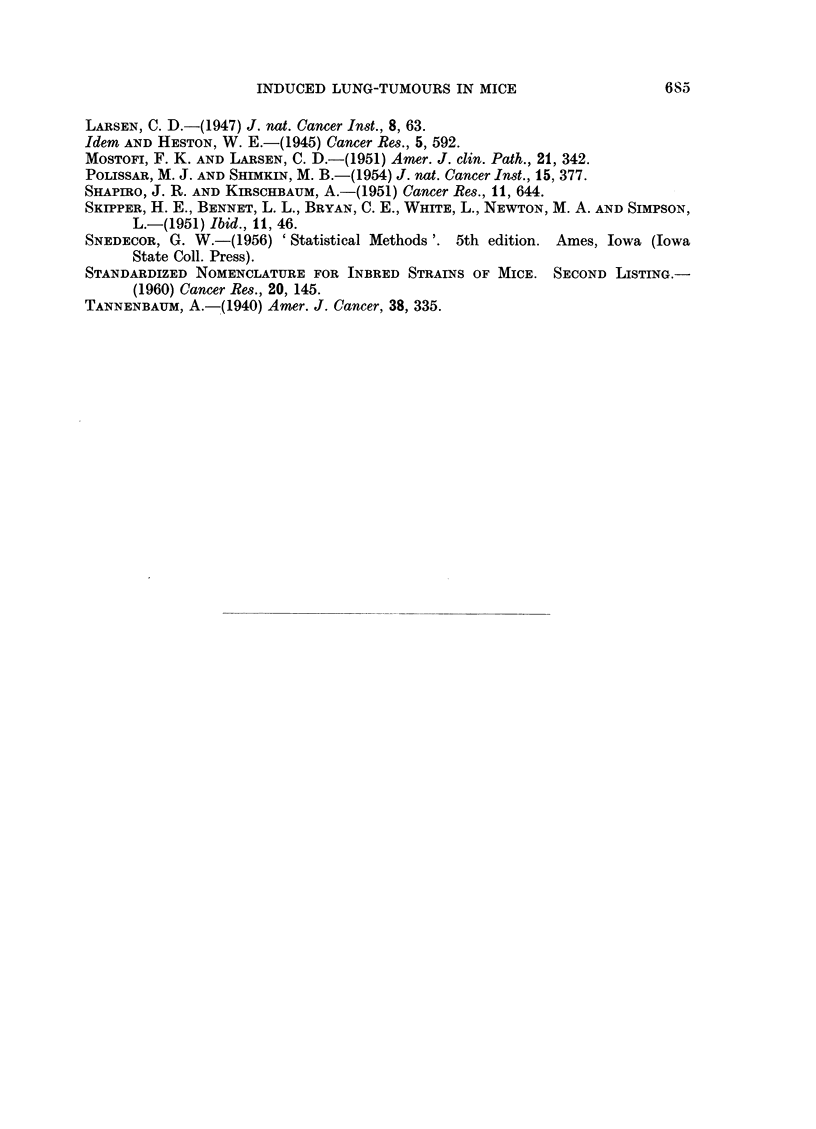# A Genetic Study of Induced Lung-Tumours in Mice

**DOI:** 10.1038/bjc.1962.77

**Published:** 1962-12

**Authors:** D. S. Falconer, Joyce L. Bloom


### A GENETIC STUDY OF INDUCED LUNG-TUMOURS IN MICE

D. S. FALCONER* AND JOYCE L. BLOOM

*Institute of Animal Genetics, Edinburgh*

Received for publication September 14, 1962

THOUGH there is now abundant proof of the importance of genetic factors in the causation of many forms of cancer (Heston, 1959), the precise mode of inheritance has proved difficult to establish, even in mice, on which many genetic studies have been made. The development of inbred strains of mice showed at once that there are genetically determined differences of susceptibility to various forms of cancer, both spontaneous and induced, since inbred strains kept under the same conditions were found to differ from each other. Genetic studies of crosses between differing strains, however, did not reveal the Mendelian ratios in the segregating $F_2$ and backcross generations that were to be expected if the inheritance was simple. The strains were thus shown to differ by more than one or even a few genes affecting susceptibility, and the inheritance was proved to be multifactorial, or quantitative (Heston, 1942*a*, *b*). This, unfortunately, makes genetic studies much more difficult, both technically and conceptually, and little progress has been made in advancing the study of cancer into the field of quantitative genetics. One technical difficulty is simply the need to study much larger numbers of individuals than are required for Mendelian analysis, but perhaps a greater one arises from the fact that it is no longer sufficient to distinguish between susceptible and non-susceptible individuals, because there are degrees of susceptibility. It is therefore necessary to find some means of measuring the degree of susceptibility. The conceptual difficulties arise because the genetic questions to be asked are of a different sort and are largely unfamiliar to those who are not specialists in this branch of genetics. Moreover, the well-known methods of genetic analysis by the study of ratios are no longer appropriate and other methods, involving more complicated statistical treatment of the data, are required.

This paper describes an analysis of urethane-induced pulmonary adenomas in mice by methods appropriate to multifactorial inheritance. A general account of these methods and the underlying principles may be found in Falconer (1960*a*). A preliminary report of part of the work was given by Falconer and Bloom (1961). Pulmonary tumours were chosen for study because the multiple tumour-nodules provide a means of measuring quantitatively the degree of susceptibility of individual mice, the number of tumours detectable at a fixed age being taken as a measure of susceptibility. It will be shown later that the number of tumours does not provide an exact measure of the " true " susceptibility, but the distinction between " observed " and " true " susceptibility need not be made at this stage.

The object was to study a random-bred strain (i.e. genetically heterogeneous, non-inbred) and to find out to what extent the differences of susceptibility be-

* Agricultural Research Council, Unit of Animal Genetics.

tween the individuals were genetically determined. Actually two random-bred strains were studied; they had different histories and, as it turned out, somewhat different tumour-characteristics. A random-bred strain is the best model of a natural population that can be achieved with an experimental animal, and the conclusions drawn about its genetic properties, though not necessarily valid for any other strain, are much more likely to be generally valid than conclusions drawn from inbred strains or their crosses, each of which represents a single, unique, genotype. Differences between the individuals of a random-bred strain can be attributed in part to the differences of genotype and in part to non-genetic causes. To assess the relative importance of genetic and non-genetic determinants of susceptibility it is necessary to study the variation of susceptibility. The variation, measured as variance, can be partitioned into components attributable to the different causes, and the relative magnitudes of these components provide a measure of the relative importance of the causes as determinants of susceptibility. The variance that is directly observable from the differences of tumour number among the individuals is the *phenotypic variance*, symbolized as $V_P$. The phenotypic variance of a random-bred strain is divisible first into two components, the *genetic variance*, $V_G$, and the *non-genetic variance*, $V_{NG}$. The proportion that the genetic variance makes of the total variance—i.e. the ratio $V_G/V_P$—measures the *degree of genetic determination* and expresses the relative importance of heredity as a determinant of susceptibility. Or, more rigorously stated, it expresses the relative importance of differences of genotype among the individuals as determinants of their differences of susceptibility under the conditions of the experiment. The partition of the variance into genetic and non-genetic components can be made experimentally if highly inbred strains, or the $F_1$ generation of crosses between highly inbred strains, are available. Since differences of genotype among the individuals of an inbred strain or an $F_1$ are negligible, the phenotypic variance contains no genetic component. The observed variance of an inbred strain or an $F_1$ cross therefore provides an estimate of the non-genetic variance, $V_{NG}$. If it can be assumed that the individuals of the random-bred strain exhibit the same amount of non-genetic variance, then the genetic variance can be obtained simply by subtraction:

Random-bred strain: $V_{P(\text{R-B})} = V_G + V_{NG}$

Inbred or $F_1$: $V_{P(\text{I})} = V_{NG}$

Difference: $V_{P(\text{R-B})} - V_{P(\text{I})} = V_G$

There is no method of estimating the genetic variance directly from the random-bred strain itself. The equivalence of non-genetic variance between inbreds and random-breds therefore needs careful scrutiny. This will be given in the appropriate section; here it need only be said that six inbred strains and the full set of 15 crosses, including reciprocals, were used for the estimation of the non-genetic variance.

There is a second subdivision of the variance, which can be made by considering the resemblance between relatives. This subdivision is important for two reasons: first, it can be made directly from observations of the random-bred strain itself; and, second, it expresses the degree to which differences of susceptibility are "inherited", in the sense of being transmitted from parents to offspring. Theoretical considerations show that two sorts of genetic variance can be distinguished

—*additive*, $V_A$, and *non-additive*, $V_{NA}$. Estimated in the manner described above, the genetic variance contains both components: $V_G = V_A + V_{NA}$. But the degree of resemblance between relatives provides an estimate of the additive genetic variance alone. The distinction between the additive and the total genetic variance is necessary for the following reason. The susceptibility of an individual, in so far as it is genetically determined, is determined by the genotype as a whole ; i.e. the paired genes at all relevant loci. But the genotype is not transmitted to the progeny ; the genes are dissociated from their partners in the gametes and the genotypes are reconstituted afresh in the progeny. Therefore the genetically determined susceptibility may not be exactly transmitted to the progeny. This discrepancy occurs when there is dominance of one gene over its allele, or when there is epistatic interaction between genes at different loci. For example, a pair of fully pigmented mice can produce some albino offspring ; though albinism and full pigmentation are wholely determined by the genotype, the progeny as a whole are not necessarily exactly like their parents.

The additive variance is the portion of the genetic variance that is associated with the transmission of genes through the gametes, and the non-additive variance is the additional variance arising from the combination of genes into particular genotypes. The proportion that the additive variance makes of the total phenotypic variance—i.e. the ratio $V_A/V_P$—is known as the *heritability*. This expresses the degree to which susceptibility is " inherited " in the sense of being transmitted from parents to offspring. If there is no dominance or epistasis there is no non-additive variance ; the additive variance is then the whole of the genetic variance, and the heritability and the degree of genetic determination are the same.

The heritability, or the amount of additive variance, can be estimated from the degree of resemblance between relatives in a random-bred strain. The regression of the mean susceptibility of offspring on the mean susceptibility of their two parents provides an estimate of the heritability. The heritability can be estimated in this way more reliably than can the degree of genetic determination by the method outlined above. Even if one is interested primarily in the degree of genetic determination, the heritability provides a useful check because the degree of genetic determination cannot be lower than the heritability. Since the estimation of the heritability is simpler as well as being more reliable, it will be described first when the results are presented.

### STRAINS AND METHODS

### *Strains used*

The two genetically heterogeneous strains studied will be referred to as JC and LX respectively. The JC-strain originated from crosses between several different non-inbred strains, and had subsequently been maintained by ten pairs of parents per generation with minimal inbreeding for 30 generations, during which time it had served as a control for a selection experiment (Falconer, 1960*b*). The LX-strain was the product of recent crosses between four strains all of which had previously been selected for large body size with minimal inbreeding. The first set of parents used here were the progeny of the $F_2$ of the crossing. Though these mice and the subsequent generations were mated at random, the strain was a newly synthesized one which had not previously been random mated. It was therefore not as good a model of a natural population as was the JC-strain.

The six inbred strains used for the estimation of non-genetic variance were the following: A/Fa, CBA/Fa, C57BL/Fa, RIII/Fa, JU/Fa, and " KL ". The last two are strains of local origin; JU is a listed strain (Standardized Nomenclature for Inbred Strains of Mice, 1960) but KL is not. The KL-strain originated from a four-way cross of the first four strains mentioned above and had subsequently been through 38 generations of brother-sister mating.

*Treatment*

All the mice received the same treatment. Young mice were weaned and vaccinated against ectromelia at 3 weeks of age. For the induction of pulmonary tumours two intraperitoneal injections of a 10 per cent solution of urethane in distilled water were given, the first at 3 weeks, when 0·1 ml. of solution was injected, and the second at 9 weeks, when 0·28 ml. of solution was injected. The dosage given was the same for all mice, irrespective of their body weight. The use of equal doses has the great advantage that males and females then have the same average number of tumours (Larsen and Heston, 1945). The mice were weighed at regular intervals so that any connection between tumour-number and body-weight under this system of dosing could be detected. The results of this analysis will be presented in a later paper. The mice were killed at 23 weeks of age. The lungs were dissected out and fixed by distension with Fekete's modification of Tellyesniczky's fluid as described by Heston and Pratt (1959), to which indian ink had been added. The tumours visible without magnification on the surface of the lungs were counted on the next day. The animals to be used as parents were mated between 3 and 5 weeks after the second injection, i.e. at 12 to 14 weeks of age. This gave them time to rear one litter, and often if necessary two litters, before autopsy. The interval of 3 weeks between the second injection and mating gave plenty of time for the elimination of the urethane since 90 per cent of the urethane administered is eliminated from the body within 24 hours (Bryan, Skipper and White, 1949; Skipper *et al.*, 1951). This precaution was necessary because urethane affects the foetuses transparentally (Larsen, 1947; Klein, 1954). The treated mice grew consistently and regularly and bred well, the average number of young weaned per litter being 7·2 in the JC-strain and 9·9 in the LX-strain. There was no evidence of the disturbance of the reproductive system or of the reproductive capacity previously reported by Mostofi and Larsen (1951). The major organs of the body were examined at autopsy for signs of abnormalities. Fewer than 5 per cent of over 2,000 animals examined had any detectable abnormality.

The age of 23 weeks, at which to count the tumours, was chosen for purely practical reasons, as being the best compromise between a high yield of tumours and a short generation interval. The conclusions drawn about the " susceptibility " measured in this way do not necessarily apply to susceptibility measured in any other way; in particular, if the mice had been left for longer before autopsy the number of tumours would presumably have been greater and the genetic properties of " susceptibility " might well have been different in detail.

*Statistical analyses*

The distribution of tumour-numbers is markedly asymmetrical, particularly when the mean tumour-number is low, and this makes a transformation of scale desirable for the purpose of statistical analysis, since most statistical analyses require a normal (symmetrical) distribution for their validity. The transformation to square roots was found to give a satisfactorily symmetrical and approximately normal distribution, and the computations were first made on the square roots of the tumour-numbers. The results given in the preliminary report (Falconer and Bloom, 1961) were based on these computations. The estimation of the non-genetic variance from the $F_1$'s and inbreds and the extrapolation to the random bred strains is, however, not entirely satisfactory when the variances are computed from the transformed data, and this part of the analysis is best made from the untransformed counts. The computations of the heritabilities were made both for untransformed tumour-numbers and for the square roots of tumour-numbers. The results of both methods will be given because the differences between them are of some genetical interest.

Except where otherwise stated the statistical analyses followed the methods described by Snedecor (1956) and need not be described here.

### RESULTS

### *Heritability*

The heritability of tumour-number, which is the additive genetic variance as a proportion of the total, phenotypic, variance, was estimated from the degree of resemblance between offspring and their parents in respect of tumour-number. Records of the tumour-numbers of parents and their offspring were obtained from two generations of the JC-strain and from three generations of the LX-strain. There were altogether 79 pairs of JC parents with their offspring and 62 pairs of LX parents with their offpsring. The mice to serve as parents were mated in single pairs at random 3–5 weeks after their second injection of urethane, and one or two litters were obtained from them before they were due for autopsy. The offspring in the first litters of each pair of parents were injected and autopsied in their turn, some having meantime been used as parents for the next generation. Second-litter offspring were treated with urethane in a few cases where the first litters failed, but were not used as parents. In the JC-strain all the offspring in the litter were treated and their tumours counted, but in the LX-strain not more than four mice per litter were treated. The numbers of offspring in the families of the JC-strain consequently varied considerably. Allowance for the differences of family size was made by a modification (Falconer, in press) of the method of weighting described by Kempthorne and Tandon (1953). This was not done for the LX-strain where the families differed little in size.

In the absence of complications, the most precise estimate of the heritability will be obtained from the regression of the mean tumour-number of the offspring on the mean tumour-number of their two parents. Complications might arise from differences between males and females in their mean tumour-numbers or in their variances of tumour-number, or from unequal inheritance from fathers and mothers arising from maternal effects or from sex-linkage. These possibilities should therefore be examined first. The comparisons of males and females given in Table I shows that the sexes do not differ either in mean or in variance. The two sexes can therefore be treated as being equivalent and need not be analysed separately. Table II shows the degree of resemblance between offspring and parents, measured by the regression coefficient, computed separately

TABLE I.—*Comparisons of Males and Females. Means and Variances (with their Standard Errors) of the Square Roots of Tumour-numbers*

None of the differences between males and females is significant.

| Sex | Number of mice | Mean | Variance |
|---|---|---|---|
| *JC-strain* | | | |
| Males | 354 | 1·65±0·046 | 0·756±0·057 |
| Females | 330 | 1·57±0·049 | 0·785±0·061 |
| *LX-strain* | | | |
| Males | 126 | 2·50±0·124 | 1·93±0·244 |
| Females | 123 | 2·53±0·131 | 2·11±0·270 |

TABLE II.—*Regression Coefficients of Offspring on Parents in the JC-strain, Computed from the Square Roots of the Tumour-numbers*

Regression coefficients for the two sexes of offspring and the two sexes of parents are shown separately, each with its standard error, the data from all generations being combined.

| Sex of offspring | Sex of parent: Male | Sex of parent: Female |
|---|---|---|
| Male | 0·24±0·06 | 0·20±0·05 |
| Female | 0·17±0·06 | 0·17±0·05 |
| Both | 0·19±0·05 | 0·19±0·045 |

for the two sexes of offspring and the two sexes of parents. The regression coefficients are as alike as could be expected in view of the sampling errors, and there is therefore no reason to think that the inheritance is complicated by any form of unequal inheritance from the two parents. Males and females can therefore be treated as being equivalent both as offspring and as parents. Throughout the rest of this paper data from males and females will be combined without distinction.

The best estimate of the heritability is to be obtained from the regression of the mean of all the offspring in a family on the mean of their two parents. The values so obtained are given in Table III, and the regression in the JC strain is

TABLE III.—*Heritability of Tumour-number Estimated by the Regression of the Mean of Offspring on the Mean of Parents*

The figures given are the regression coefficients (± standard errors) calculated within generations and pooled over generations. *N* is the total number of families.

| Strain | *N* | Tumour-numbers (untransformed) | Square roots of tumour-numbers |
|---|---|---|---|
| JC | 79 | 0·232±0·048 | 0·323±0·051 |
| LX | 62 | 0·487±0·135 | 0·545±0·148 |

illustrated in Fig. 1. The heritability of tumour-number (untransformed) is 23 per cent in the JC-strain and 49 per cent in the LX-strain. This means that in the JC-strain, for example, about one quarter of the variability of tumournumber is inherited in the sense of being transmitted from parents to offspring. In other words about one quarter of the difference of tumour-number between two pairs of parents reappears as a difference between their offspring: in the LX-strain about one half of the parental differences are transmitted. The difference of heritability between the two strains is not quite significant at the 5 per cent level.

Computations made from the square roots of the tumour-numbers yielded higher estimates of the heritability, particularly in the JC-strain where the

FIG. 1.—Resemblance between offspring and their parents in respect of tumour-number in the JC-strain. The parents are grouped according to the parental mean, i.e. the mean tumour-number of the mated pair, and the points show the mean tumour-number in all the offspring from each parental mean. The straight line is the regression of offspring on parental mean, fitted by least squares. Its slope, 0·23 $\pm$ 0·05, measures the heritability of tumour-number.

value of 32 per cent was obtained in place of 23 per cent calculated from the untransformed tumour-numbers. The reason for the difference is connected with the asymmetrical distribution of tumour-numbers, which was greater in the JC-strain. The LX-strain, with less asymmetry in the distribution, showed a smaller difference in the heritability—54 per cent computed from square roots in place of 49 per cent computed from untransformed tumour-numbers. The higher heritability of the square roots of tumour-numbers means that the square root is better than the number itself as an indication of an individual's potentiality for transmitting higher or lower tumour-number to its offspring. The genetic significance of this will be discussed later.

Determination of the heritability leads to the partitioning of the phenotypic variance into two parts. The amount of additive genetic variance is determined by multiplying the phenotypic variance by the heritability. The remainder of the variance is the non-additive genetic together with all the non-genetic variance. This partitioning of the variance of (untransformed) tumour-numbers is given in Table IV. The further partitioning of the remainder is the subject of the next section.

TABLE IV.—*Partitioning of the Variance of Tumour-numbers (Untransformed) by the Heritability*

| Component of variance | Strain JC | Strain LX |
|---|---|---|
| Phenotypic (= total) $V_P$ | 8·50 | 51·92 |
| Additive genetic $V_A$ | 1·97 | 25·28 |
| Remainder $V_{NA} + V_{NG}$ | 6·53 | 26·64 |

*Estimation of non-genetic variance*

The amount of variation arising from non-genetic causes among the individuals of a random-bred strain cannot be estimated by observations of the random-bred strain itself: it can only be inferred from observations of genetically uniform strains. To draw conclusions about one group of animals from observations made on another has obvious dangers and careful scrutiny of the procedures adopted will have to be made. The phenotypic variance among the individuals of one inbred strain, or of the $F_1$ cross between two inbred strains, provides a measure of the non-genetic variation exhibited by that particular genotype. There is, however, no *a priori* reason to suppose that other genotypes will exhibit the same amount of non-genetic variation, because some genotypes may be more sensitive to environmental effects than others. Therefore the first prerequisite is that the estimate to be used should be derived from a large enough number of different inbreds or $F_1$s to give a reasonable representation of the array of genotypes likely to exit in the random-bred strain. Furthermore, inbred strains do not represent genotypes that could occur in a random-bred strain because the inbred genotypes are completely homozygous. Therefore $F_1$ crosses are to be preferred for the estimation of non-genetic variation, and inbred strains can only be used if they can be shown not to differ consistently from $F_1$s in variability.

In this experiment estimates of the variance of tumour-number were obtained from six inbred strains and the 15 crosses between them. Reciprocal crosses were made but there were no significant differences in mean tumour-number between them so the data from the reciprocals were combined. The inbreds as a whole did not differ from the crosses, either in mean or in variance, so the data from the inbreds as well as from the crosses were used. Thus the final estimate of the non-genetic variance was based on the variability exhibited by 21 different genotypes. This gives some assurance that the estimate is likely to be representative of the non-genetic variance exhibited by the individuals of the random-bred strains. There were altogether 723 animals, with mostly between 30 and 40 in each group. The mean tumour-number and the variance of tumour-number of each strain and cross are given in Table V. Each variance is, of course, the variance within that strain or cross, the variance between the groups being irrelevant in the present context. There were no significant differences between families or litters of the same strain or cross, so individuals were combined without regard to their family or litter in the computation of the means and variances.

TABLE V.—*Tumour-numbers (Untransformed) in the Inbred Strains and Crosses*

$N$ is the number of animals. $k^2$ is estimated as (variance − mean)/(mean)$^2$. Its standard error is derived from a formula for the sampling variance of $1/k^2$ given by Bliss (1953).

| Strain or cross | $N$ | Mean | Variance | $k^2 \times 100$ | S.E. of $k^2 \times 100$ |
|---|---|---|---|---|---|
| A | 16 | 23·69 | 141·30 | 20·96 | 4·44 |
| CBA | 40 | 2·25 | 3·89 | 32·30 | 12·15 |
| C57BL | 35 | 0·91 | 0·90 | −1·20 | 29·11 |
| RIII | 38 | 11·55 | 21·17 | 7·21 | 3·94 |
| JU | 39 | 13·10 | 23·52 | 6·07 | 3·65 |
| KL | 32 | 18·84 | 35·04 | 4·56 | 3·42 |
| A × CBA | 38 | 9·40 | 19·00 | 10·88 | 4·41 |
| A × C57 | 34 | 19·53 | 54·68 | 9·22 | 3·28 |
| A × RIII | 38 | 22·68 | 93·52 | 13·77 | 2·93 |
| A × JU | 35 | 22·74 | 51·20 | 5·50 | 3·05 |
| A × KL | 35 | 20·31 | 50·93 | 7·42 | 3·19 |
| CBA × C57 | 34 | 3·65 | 3·87 | 1·69 | 8·89 |
| CBA × RIII | 31 | 5·19 | 7·16 | 7·29 | 7·16 |
| CBA × JU | 34 | 5·12 | 5·68 | 2·16 | 6·91 |
| CBA × KL | 33 | 5·21 | 5·36 | 0·55 | 6·92 |
| C57 × RIII | 36 | 10·97 | 23·69 | 10·56 | 3·78 |
| C57 × JU | 36 | 7·25 | 6·82 | −0·82 | 5·31 |
| C57 × KL | 40 | 11·95 | 14·97 | 2·12 | 3·77 |
| RIII × JU | 40 | 11·63 | 35·83 | 17·91 | 3·83 |
| RIII × KL | 24 | 13·96 | 20·91 | 3·57 | 4·51 |
| JU × KL | 35 | 15·91 | 17·08 | 0·46 | 3·52 |

A glance at the values in Table V shows at once that the groups differ widely in variance, and, further, that the variance is strongly correlated with the mean ; groups with high means have in general high variances. It is therefore necessary to take account of the mean tumour-number of each group and to find some measure of variability that is independent of the mean. This measure can then be applied to the random-bred strains to yield estimates of the non-genetic variance most likely to be associated with the particular mean of each. The estimate appropriate to the JC-strain in the preliminary report (Falconer and Bloom, 1961), which referred to the square roots of tumour-numbers, was based on a purely empirical method of adjusting for differences of mean. Further consideration convinced us that this method was not entirely satisfactory and that it is necessary to work with the untransformed tumour-numbers. It then becomes possible to derive a measure of variability that is independent of the mean. This measure, which is essentially a coefficient of variation, has a simple theoretical basis and a clear biological meaning. In explanation of the method to be applied it is necessary first to consider the nature of the variation of tumour-number and the distinction between " tumour-number " and " susceptibility ".

In an illuminating study of the variation of tumour-numbers, Polissar and Shimkin (1954) arrived at the following conclusions about the nature of the variation. Any individual mouse has a certain " susceptibility ", but the number of tumours it forms is not necessarily precisely characteristic of its particular susceptibility, because there is an element of chance in whether a cell becomes tumorous or not. Thus, if it were possible to have a number of mice, identical in every biological respect, so that they all had exactly the same susceptibility, they would still differ in the number of tumours they developed. The variation among such a group of mice would be ascribable only to chance as a " cause " and it would be expected to follow the well-known Poisson distribution, which has the characteristic that the variance is numberically equal to the mean. The variance of tumour-number among a (hypothetical) group of identical mice would therefore be expected to be equal to the mean tumour-number. In any group of real mice the differences of susceptibility cause additional variation superimposed on the variation arising from chance. Subtraction of the variance expected from chance, i.e. the mean tumour-number, from the observed variance of tumour-number therefore provides a measure of the variance of susceptibility.

This analysis divides the total variance of tumour-number into two components, variance of susceptibility and variance due to chance, a division which cuts across the division into genetic and non-genetic variance. In a genetic analysis the variance due to chance will, of course, all appear as non-genetic variance. The variance of susceptibility among inbred or $F_1$ mice will also be non-genetic, so there are two sorts of non-genetic variance, chance variance, for which the symbol $V_C$ will be used, and non-genetic variance of susceptibility. We shall refer to the non-genetic variance of susceptibility as " environmental variance ", with the symbol $V_E$, on the supposition that the cause of this variation is primarily the environmental variables operating over the course of the experiment. The environmental variance thus embraces all real differences of susceptibility that are non-genetic in origin, as well as differences of apparent susceptibility caused by technical " errors " such as small differences in the doses of carcinogen administered. The genetic differences in a random-bred strain produce additional variance of susceptibility, so the variance of susceptibility in a random-bred strain will be partly genetic and partly non-genetic. The dual classification of the variance of tumour-numbers into genetic *versus* non-genetic and susceptibility *versus* chance may be symbolized thus :

$$\text{Observed variance of tumour numbers} = \underbrace{\overset{\text{Genetic}}{V_G} + \overbrace{V_E}^{\text{Non-genetic}}}_{\text{Susceptibility}} \overbrace{+ \underbrace{V_C}_{\text{Chance}}}$$

Polissar and Shimkin (1954) found that when groups of mice of the same strain were subjected to different dosages of carcinogen under the same general treatment, then the variance of susceptibility was proportional to the square of the mean tumour-number. The coefficient of variation (standard deviation/mean) was thus constant, and suceptibility was found to follow a relationship common among biological variables. The variance of susceptibility can therefore be written as $k^2m^2$, where $m$ is the mean tumour-number and $k$ is the coefficient of variation characteristic of the strain and treatment. By adding the chance variance, taken as equal to the mean, the observed variance of tumour-number in any group of mice can be written in the form $V = m + k^2m^2$. The data analysed by Polissar and Shimkin were entirely consistent with this interpretation of the nature of the variation, and our data also fit it well. The equation expressing the interpretation can be applied to the solution of the present problem with full confidence that it adequately describes the relationship between variance and mean. If the value of $k^2$ is first calculated from the inbred and $F_1$ data, then the non-genetic variance in the random-bred strains can be evaluated from the equation $V = m + k^2m^2$ by putting $m$ equal to the mean tumour-number of the random-bred strain. It will, however, be more informative to keep the terms $m$ and $k^2m^2$ separate since $m$ evaluates the chance variance, $V_C$, and $k^2m^2$ evaluates the environmental variance, $V_E$, in the random-bred strain.

FIG. 2.—Values of $k^2$ for each of the 21 inbred strains or $F_1$ crosses plotted against the mean tumour-number, to show that $k^2$ is independent of the mean, and that the inbred strains as a whole did not differ from the $F_1$ crosses.

Let us return to the inbred and $F_1$ data given in Table V. The squared coefficient of variation, $k^2$, was calculated from each strain and cross as

$$k^2 = \frac{V - m}{m^2},$$

where $V$ is the observed variance of tumour-number and $m$ is the mean tumour-number in the strain or cross. The values of $k^2$ are given in Table V. They vary over quite a wide range and before proceeding it is necessary to verify that $k^2$ is really independent of the mean. Fig. 2 shows the value of $k^2$ plotted against the mean tumour-number for each strain and cross. Though there is a slight tendency for higher values of $k^2$ to be associated with higher means, the computed regression of $k^2$ on $m$ was not significantly different from zero ($P > 0{\cdot}1$). It is therefore justifiable to proceed on the assumption that $k^2$ is independent of the mean. We may note in passing that Fig. 2 shows also that the inbred strains as a whole do not differ from the crosses in their values of $k^2$; this is the justification for including the inbred strains in the estimate of the non-genetic variance.

To arrive at an overall estimate of the " average " value of $k^2$ is not altogether simple because the individual estimates differ widely in precision, and the weighting factor appropriate to the precision depends in a rather complicated manner on the mean tumour-number and the number of animals in the group. The procedure followed for obtaining an overall estimate of $k^2$ with appropriate weighting was that described by Bliss and Owen (1958). For details of the method the reader must be referred to the paper cited. The mean value of $k^2$ obtained by this method was

$$\overline{k^2} = 0{\cdot}0788 \pm 0{\cdot}0130$$

with approximate 95 per cent confidence limits of 0·052 and 0·106. The method also provides a test of whether the groups differ significantly in their values of $k^2$. This test of heterogeneity showed that the observed differences of $k^2$ between the strains and crosses could not have arisen from sampling errors ($P = 0{\cdot}01$). It must therefore be concluded that the strains and crosses are not all alike in their coefficient of variation of susceptibility. Despite the heterogeneity it seems most appropriate to use the weighted mean of $k^2$ because the precision of the separate estimates differs so widely. (A further test of interest provided by the analysis was whether the variance tended to zero as the mean tended to zero, which is a necessary condition for the validity of the equation $V = m + k^2m^2$, on the basis of which the non-genetic variance of the random-bred strains is to be deduced. This condition was satisfied.)

*Components of variance of the random-bred strains*

The overall estimate of $k^2 = 0{\cdot}0788$ can now be used to deduce the environmental variance of susceptibility expected in the random-bred strains. This will be given by $k^2m^2$, where $m$ is the mean tumour-number of the random-bred strain. The total variance of tumour-number can then be partitioned into the three components

$$V_P = V_C + V_E + V_G$$

where $V_C$ is the variance attributable to chance, which is equal to $m$; $V_E$ is the environmental variance of susceptibility and is equal to $k^2m^2$; and $V_G$ is the genetic variance of susceptibility, which is obtained as a remainder. The genetic variance can be subdivided into additive and non-additive portions since the additive genetic variance has already been estimated (Table IV). The means and variances of the two random-bred strains are given in Table VI, and the partitioning of the variance is shown in Table VII. Table VIII gives the compo-

TABLE VI.—*Means and Variances of (Untransformed) Tumour-numbers in the Two Random-bred Strains, with their Standard Errors*

| | JC-strain | LX-strain |
|---|---|---|
| Number of mice | 684 | 249 |
| Mean | 3·42±0·11 | 8·19±0·46 |
| Variance | 8·50±0·46 | 51·92±4·66 |

nents as percentages of the total and thus shows the relative importance of the different causes of variation of tumour-number.

TABLE VII.—*Partitioning of the Variance of Tumour-number (Untransformed) in the Random-bred Strains*

| Component of variance | | JC-strain | LX-strain |
|---|---|---|---|
| Phenotypic (= total) | $V_P$ | 8·50 | 51·92 |
| Chance (= mean, $m$) | $V_C$ | 3·42 | 8·19 |
| Environmental ($=k^2m^2$) | $V_E$ | 0·92 | 5·28 |
| Total genetic ($= V_P - V_C - V_E$) | $V_G$ | 4·16 | 38·45 |
| Additive genetic (from Table IV) | $V_A$ | 1·97 | 25·28 |
| Non-additive genetic ($= V_G - V_A$) | $V_{NA}$ | 2·19 | 13·17 |

TABLE VIII.—*Percentage Composition of the Variance of Tumour-numbers (Untransformed) in the Random-bred Strains*

The figures in brackets are approximate 95 per cent confidence limits.*

| Component of variance | | | JC-strain | | | LX-strain | | |
|---|---|---|---|---|---|---|---|---|
| Additive genetic | $V_A$ | $V_G$ | 23 | 49 | (43–54) | 49 | 74 | (68–78) |
| Non-additive genetic | $V_{NA}$ | | 26 | | | 25 | | |
| Environmental | $V_E$ | | 11 | | (7–15) | 10 | | (7–14) |
| Chance | $V_C$ | | 40 | | (38–43) | 16 | | (14–18) |
| | | | 100 | | | 100 | | |

* Derivation of confidence limits :

$V_G$ : from $\pm$ 2 standard errors of the estimate of the total variance (Table VI), sampling errors of $V_E$ and $V_C$ being neglected.

$V_E$ : from the confidence limits of $k^2$ given in the text, the sampling error of $m$ being neglected.

$V_C$ : from $\pm$ 2 standard errors of the estimate of the mean (Table VI).

The variances of the inbred strains and crosses, and the partitioning of the variances of the random-bred strains to which they lead, are illustrated graphically in Fig. 3. The variance of each strain and cross is shown as a point plotted against the mean tumour-number of that strain. A straight line is drawn showing the variance attributable to chance at any level of mean ($V_C = m$), and a curved line is drawn showing the average variance of the inbreds and crosses appropriate to any mean, as $V = m + 0{\cdot}0788\ m^2$. The variances of the two random-bred strains are shown as vertical bars extending to the appropriate height, so that the partitioning into variance due to chance and environmental variance of susceptibility is shown by the intersections. The percentage compositions of the variances are shown diagrammatically in Fig. 4.

The approximate 95 per cent confidence limits to the percentages, given in Table VIII, give at least a rough idea of the statistical reliability of the partitioning of the variance into genetic, environmental and chance components. They show that sampling errors are not large enough to alter materially the general conclusions about the relative importance of the different sources of variation.

*Degree of genetic determination*

The chief object of the analysis of the inbred strains and crosses was to deduce the degree of genetic determination of tumour-number in the random-bred strains —i.e. the amount of genetic variance as a proportion of the total. As shown in

FIG. 3.—Graphical representation of the variance of the inbred and $F_1$ mice and the partitioning of the variance of the random-bred strains, as explained in the text.

FIG. 4.—The complete partitioning of the variances of the two random-bred strains, with the separate components as percentages of the total, to show the relative importance of each source of variation.

Table VIII this was 49 per cent in the JC-strain and 74 per cent in the LX-strain This means that genetic and non-genetic factors were about equally important as determinants of the differences of tumour-number among the individuals of the JC-strain, while genetic factors were about three times as important as non-genetic factors in the LX-strain. The striking difference between the two strains in the degree of genetic determination is, however, no more than a reflection of the difference of mean tumour number, and results from the contribution of chance to the variance. The variation due to chance increases in proportion to the mean tumour-number, while the variation of susceptibility increases as the square of the mean. Consequently chance contributes proportionately less variance when the mean is high than when it is low, as may be seen from Fig. 3. A more meaningful comparison of the two strains can therefore be made by excluding the variation due to chance and considering only the variation of true susceptibility. The percentage composition of the variation of susceptibility is shown in Table IX. The two strains are now seen to be much more alike. Differences of genotype among the individuals account for 82 per cent of the variation of susceptibility in the JC-strain and 88 per cent in the LX-strain, while environmental differences account for 18 and 12 per cent respectively. Though it is

TABLE IX.—*Percentage Composition of the Variance of Susceptibility (i.e. Excluding Chance Variation of Tumour-number) in the Random-bred Strains*

| Component of variance | JC-strain | | LX-strain | |
|---|---|---|---|---|
| Additive genetic | 39 | 82 | 58 | 88 |
| Non-additive genetic | 43 | | 30 | |
| Environmental | 18 | | 12 | |

difficult to attach standard errors to these figures it seems probable that sampling errors could easily account for this difference between the strains. We therefore have no reason to suppose that the two random-bred strains differ from each other in the relative importance of genetic and environmental differences as determinants of susceptibility, though in consequence of their different mean susceptibilities they do differ in respect of the variation of tumour-number. The heritability of tumour-number was shown in an earlier section to be higher in the LX-strain than in the JC-strain, and this is what would have been expected from the smaller contribution of chance in the LX-strain with its higher mean. Removal of the chance variance still leaves the heritability of susceptibility higher in the LX-strain (58 per cent) than in the JC-strain (39 per cent), as shown in Table IX. But since the original difference, with chance included, was not significant, the smaller difference, with chance removed, does not necessarily represent a real difference between the strains.

*Non-additive genetic variance and the sib-correlation*

The figures of 49 per cent and 74 per cent obtained for the degree of genetic determination of tumour-number are considerably higher than the heritabilities determined from the resemblance between relatives, which were 23 and 49 per cent respectively. Since the heritability expresses the amount of additive genetic variance as a proportion of the total phenotypic variance, it follows that an appreciable amount of the genetic variance in both strains was non-additive. The partitioning given in Tables VII and VIII shows that about half of the genetic variance was non-additive in the JC-strain and about one-third in the LX-strain. The sources of non-additive genetic variance are dominance or partial dominance at the individual loci and epistatic interaction between the effects of genes at different loci. Confirmation of the existence of non-additive variance and an indication that dominance is probably the major source of it is provided by the correlation between full sibs. In the absence of complications this correlation is expected to be equal to half of the heritability, but it will be greater than this if there are environmental causes of resemblance between sibs, such as maternal effects, or if there is an appreciable amount of non-additive variance. It is the variance arising from dominance rather than from epistasis that makes the greatest contribution to the correlation. If, therefore, we assume that all the non-additive variance arises from dominance we can arrive at an expected value of the sib-correlation for comparison with the observed value. The covariance of full sibs is made up of half the additive variance together with one-quarter of the dominance variance, so the expected correlation will be half the heritability plus one-quarter of the proportionate amount of non-additive variance. In the JC-strain, for example, the expected correlation will be $(\frac{1}{2} \times 0{\cdot}23) + (\frac{1}{4} \times 0{\cdot}26) = 0{\cdot}18$. The observed and expected correlations are given in Table X. The observed correlation in the JC-strain was computed

TABLE X.—*Correlations between Full Sibs (Litter-mates) in the Random-bred Strains, Computed from (Untransformed) Tumour-numbers*

| Strain | Degrees of freedom: Between families | Degrees of freedom: Within families | Observed correlation ± S.E. | Expected correlation* |
|---|---|---|---|---|
| JC | 78 | 440 | 0·20±0·05 | 0·18 |
| LX | 66 | 182 | 0·27±0·07 | 0·31 |

* Explained in text.

from the same families that provided the offspring-parent regression ; the correlation in the LX-strain was computed from two generations of which only the second contributed to the offspring-parent regression. In all cases the families of full sibs were litter-mates. The observed and expected values agree well in both strains, the sampling errors being quite enough to account for the discrepancies. The sib-correlations are therefore entirely consistent with the amount of non-additive genetic variance deduced from the difference between the heritability and the degree of genetic determination, on the assumption that the non-additive variance arose principally from dominance rather than from epistasis. We can conclude further, that there were no environmental causes of resemblance in tumour-number between litter-mates, since the genetic causes of resemblance are enough to account for the observed correlations.

Consideration of the non-additive genetic variance in the random-bred strains leads to the conclusion that some, at least, of the genes are dominant, or partially dominant, in their effects on tumour-number, though it gives no clue as to whether the dominance is for higher or for lower tumour-number. The data from the inbred strains and crosses, however, show that there was no preponderance of dominance in one direction among the genes in the inbred strains, because the overall mean of the crosses was very little different from that of the parent inbreds ; the unweighted mean of the $F_1$'s was 12·4 and that of the inbreds was 11·7 tumours. It is a reasonable inference that the genes in the random-bred strains were also dominant some in one direction and some in the other.

The asymmetrical distribution of the untransformed tumour-numbers, on which the computations were based, suggests that there will be some dominance associated with the scale of measurement, in the following way. Suppose, for example, that the genotypes $AA$, $Aa$ and $aa$ at one gene-locus had average tumour-numbers of 4, 9 and 16 respectively. The $A$-gene would then be partially dominant in the direction of low tumour-number, because the heterozygote has a value 9 while the average of the two homozygotes is 10. If, however, the tumour-numbers were transformed to square-roots the values of the three genotypes would be 2, 3 and 4, and there would now be no dominance. Thus partial dominance can be modified by a change of scale. Dominance associated in this way with the scale, and the non-additive variance to which it gives rise, would tend to disappear if the computations were made after transformation to a scale that rendered the distribution more nearly symmetrical, such as a square-root transformation. The fact noted earlier that the heritability was higher when computed from the square roots of tumour-numbers than when computed from the tumour-numbers themselves is in agreement with this expectation. The difference of heritability implies that a lesser amount of the genetic variance was non-additive when susceptibility was measured on a scale of square-roots, and therefore that there was less dominance. It does not seem likely, however, that more than a small part of the dominance effects and the non-additive genetic variance of untransformed tumour-numbers can be attributed to a " scale-effect " in this way.

### DISCUSSION

The separation of chance from the other sources of variation is an important concept in the interpretation of the variation of tumour-numbers. It leads to the distinction between the " susceptibility " of an individual and the number of tumours counted, which may differ from the susceptibility as a result of chance. Thus the number of tumours is not an exact measure of the susceptibility of an individual, though the mean number of tumours measures the mean susceptibility of a strain or group with an exactness that depends only on the number of animals whose tumours are counted. While the experimental observations are necessarily limited to the number of tumours, it is the variation of susceptibility that carries the greater interest since the variation arising from chance is in a sense biologically irrelevant. Because the variation due to chance and the variation of susceptibility alter with the mean tumour-number in different ways, chance plays a relatively smaller role when the mean is high than when it is low. Consequently comparisons, between strains or experiments, of results referring to the variation of tumour-numbers are not very meaningful, but comparisons of results referring to susceptibility are.

Two comparisons of our results can be made with previous work, and in both respects they agree well. Heston (1942*a*) studied the variation of dibenzanthracene-induced lung tumours in the generations following a cross between two inbred strains of widely different susceptibilities. By comparing the variance of tumour-numbers in the segregating $F_2$ generation with that of the genetically uniform $F_1$ he concluded that about 86 per cent of the variance in the $F_2$ was genetically determined. This is what we have called the degree of genetic determination of tumour-number. The degree of genetic determination of susceptibility, which can be deduced by taking account of the mean tumour-number, was 95 per cent in Heston's experiment. Our values for the two random-bred strains were 82 per cent and 88 per cent respectively. Though Heston's value probably does not differ significantly from ours, a higher degree of genetic determination would be expected in an $F_2$ of a cross between widely divergent strains than in a random-bred strain, because all the genes responsible for the wide difference segregate in the $F_2$. The close agreement between the three estimates justifies the general conclusion that susceptibility to the induction of lung tumours has a very high degree of genetic determination. That genetic differences can be the cause of large differences of susceptibility has, of course, been known for a long time from the wide differences between inbred strains. Our experiment proves that the wide range of genetic diversity is not confined to differences between inbred strains, but that among the individuals of random-bred strains, which are the nearest laboratory equivalent of natural populations, genetic differences are by far the most important cause of differences of susceptibility.

The second comparison that can be made refers to the amount of environmental variance of susceptibility, for which the appropriate measure is the coefficient of variation of susceptibility, $k$, in inbred strains or $F_1$ crosses. One cannot expect different experiments necessarily to yield the same coefficient of variation, especially if made with different strains, because the techniques of induction may differ in various ways, and our data showed that the inbred strains and crosses differed significantly among themselves. The comparison is nevertheless useful in showing that our experiment did not differ in any marked way from the others. Polissar and Shimkin's (1954) analyses of four different experiments with A-strain inbreds gave values of $k$ ranging from 0·31 to 0·56. The value of $k$ in our A-strain mice was 0·46, and the overall average of all inbred strains and crosses was 0·28, with approximate 95 per cent confidence limits of 0·23 and 0·33. Heston's data referred to above, it may be added, give a value of 0·31 in the $F_1$ generation. These comparisons show that our experiment was very like the others in the amount of environmental variation exhibited.

The high degree of genetic determination, with the correspondingly small proportion of environmental variance, may seem to suggest that susceptibility is unusually insensitive to environmental influences. In our preliminary publication (Falconer and Bloom, 1961) we concluded tentatively that environmental variation accounted for only 2 per cent of the total variation of tumour-number in the JC-strain, a figure that seemed too low to be readily credible. By the analysis presented here, which we believe to be more exact, environmental sources contributed 11 per cent of the total variance of tumour-number and 18 per cent of the variance of susceptibility. Though now quite credible, this revised estimate is still small and the general conclusion remains that environmental factors are relatively unimportant. Whether susceptibility is unusually insensitive to environmental factors can only be judged, however, from the actual amount, and not from the relative amount, of environmental variance. The actual amount of environmental variance of susceptibility is not particularly small, and with a coefficient of variation of about 30 per cent it is well within the range commonly found with biological variables. To give just two examples from inbred or $F_1$ mice : body-weight at various ages had coefficients ranging from 4 to 11 per cent (Chai, 1957), and various measures of reproductive performance had coefficients ranging from 7 to 53 per cent (Barnett and Coleman, 1960). Thus there is no ground for supposing that susceptibility to the induction of lung tumours is unusually insensitive to environmental differences of the sort that occur in experimental material.

What the environmental variables that differentiate the experimental mice may be is not easy to suggest. The number of tumours is, of course, very sensitive to the dosage of carcinogen and some of the variation appearing in the analysis as environmental variance of susceptibility must undoubtedly have been caused by accidental differences in the amount of urethane administered or in the amount reaching the sites of tumour formation in the lungs. How much of the variation can be attributed to this source is not known, but it does not seem likely to be a large part. Transplantation studies (Heston and Dunn, 1951 ; Shapiro and Kirschbaum, 1951) have shown that the site of action of the genes responsible for the genetically determined susceptibility to carcinogen is in the lung tissue itself, and that the tissue-specificity is already present in foetal lungs (Heston and Steffee, 1957). The tissue-specificity suggests that the operative environmental factors are likely to be those that affect the lung tissue directly, such as the oxygen concentration, which has been proved to influence the number of tumours (Heston and Pratt, 1956, 1959 ; Dipaolo, 1957). On the other hand, restriction of food intake has also been shown to influence the number of tumours (Tannenbaum, 1940), and this suggests that the physiological state of the individual may have some effect, perhaps through the rate at which the carcinogen is eliminated from the body. Differences of physiological state associated with the state of activity of the individual at the time of administration of the urethane may therefore have been a source of the observed environmental variation.

The fact that genetic differences are so much more important than environmental factors as determinants of susceptibility does not necessarily mean that a very large number of gene-loci contribute to the genetic variation in random-bred strains. The large amount of genetic variance observed may equally well have been caused by a few genes with large individual effects or by many genes with small individual effects. There is no way of arriving at an estimate of the number of genes that cause the variation in a random-bred strain, because the number of genes cannot be separated from the magnitude of their individual effects. There are probably all degrees of gene-effects, part of the variance coming from a few genes with relatively large effects and the remainder from an indefinitely large number of genes with smaller effects.

### SUMMARY

The inheritance of susceptibility to the carcinogenic action of urethane was investigated by the genetical methods appropriate to multifactorial inheritance. The number of pulmonary adenomas induced by a standard dose of urethane was taken as a measure of susceptibility and the variation among the individuals of two random-bred strains was apportioned to genetic and non-genetic causes.

The main conclusion was that genetic differences were by far the most important cause of the differences of susceptibility among the individuals of both strains.

The results, in more detail, were as follows :

(i) There was no sex-difference in tumour-number, and the inheritance was equal from male and female parents.

(ii) The heritability of tumour-number, estimated from the resemblance between offspring and their parents, was 23 per cent in one strain and 49 per cent in the other.

(iii) Recognition of an amount of variance, numerically equal to the mean, as being attributable to chance led to the distinction between the observed tumour-number and the true susceptibility. The environmental variance of susceptibility was estimated from six inbred strains and the fifteen $F_1$ crosses between them. It had a coefficient of variation of 28 per cent. With a coefficient of variation of this magnitude, susceptibility was not unusually insensitive to environmental differences of the sort that occur in experimental material.

(iv) On the assumption that the environmental variance in the random-bred strains had the same coefficient of variation as the inbred strains and crosses, it was concluded that the degree of genetic determination of susceptibility was 82 per cent in one random-bred strain and 88 per cent in the other. This means that genetic differences among the individuals were responsible for 80–90 per cent of the variation of susceptibility.

We are grateful to the Medical Research Council for the provision of a grant which enabled this work to be done ; also to Dr. G. M. Jolly and Dr. B. Woolf for statistical advice, to Dr. W. E. Heston for advice about the method of tumour-induction, and to Dr. R. C. Roberts for criticism of the manuscript.

### REFERENCES

Barnett, S. A. and Coleman, E. S.—(1960) *Genet. Res.*, **1**, 25.
Bliss, C. I.—(1953) *Biometrics*, **9**, 176.
*Idem* and Owen, A. R. G.—(1958) *Biometrika*, **45**, 37.
Bryan, C. E., Skipper, H. E. and White, L. Jr.—(1949) *J. biol. Chem.*, **177**, 941.
Chai, C. K.—(1957) *Amer. Nat.*, **91**, 49.
Dipaolo, J. A.—(1957) *J. nat. Cancer Inst.*, **23**, 535.
Falconer, D. S.—(1960*a*) ' Introduction to Quantitative Genetics '. Edinburgh (Oliver & Boyd).—(1960*b*) *J. cell. comp. Physiol.*, **56**, Suppl. 1, 153. In ' Methodology in Mammalian Genetics '. Edited by W. J. Burdette. San Francisco (Holden-day). In press.
*Idem* and Bloom, J. L.—(1961) *Nature, Lond.*, **191**, 1070.
Heston, W. E.—(1942*a*) *J. nat. Cancer Inst.*, **3**, 69.—(1942*b*) *Ibid.*, **3**, 79.—(1959) ' Genetics and Cancer '. Austin (University of Texas), p. 226.
*Idem* and Dunn, T. B.—(1951) *J. nat. Cancer Inst.*, **11**, 1057.
*Idem* and Pratt, A. W.—(1956) *Proc. Soc. exp. Biol., N.Y.*, **92**, 451.—(1959) *J. nat. Cancer Inst.*, **22**, 707.
*Idem* and Steffee, C. H.—(1957) *Ibid.*, **18**, 779.
Kempthorne, O. and Tandon, O. B.—(1953) *Biometrics*, **9**, 90.
Klein, M.—(1954) *Cancer Res.*, **14**, 438.

LARSEN, C. D.—(1947) *J. nat. Cancer Inst.*, **8**, 63.
*Idem* AND HESTON, W. E.—(1945) *Cancer Res.*, **5**, 592.
MOSTOFI, F. K. AND LARSEN, C. D.—(1951) *Amer. J. clin. Path.*, **21**, 342.
POLISSAR, M. J. AND SHIMKIN, M. B.—(1954) *J. nat. Cancer Inst.*, **15**, 377.
SHAPIRO, J. R. AND KIRSCHBAUM, A.—(1951) *Cancer Res.*, **11**, 644.
SKIPPER, H. E., BENNET, L. L., BRYAN, C. E., WHITE, L., NEWTON, M. A. AND SIMPSON, L.—(1951) *Ibid.*, **11**, 46.
SNEDECOR, G. W.—(1956) 'Statistical Methods'. 5th edition. Ames, Iowa (Iowa State Coll. Press).
STANDARDIZED NOMENCLATURE FOR INBRED STRAINS OF MICE. SECOND LISTING.—(1960) *Cancer Res.*, **20**, 145.
TANNENBAUM, A.—(1940) *Amer. J. Cancer*, **38**, 335.